# Development steps of multimodal exercise interventions for older adults with multimorbidity: A systematic review

**DOI:** 10.1002/hsr2.2190

**Published:** 2024-07-01

**Authors:** Faye Forsyth, Chien Lin Soh, Natasha Elks, Helen Lin, Kris Bailey, Rosalie Brooman‐White, Scott Rowbotham, Jonathan Mant, Peter Hartley, Christi Deaton

**Affiliations:** ^1^ Primary Care Unit, Department of Public Health and Primary Care University of Cambridge Cambridge UK; ^2^ KU Leuven Department of Public Health and Primary Care KU Leuven Belgium; ^3^ University of Cambridge School of Clinical Medicine Cambridge UK; ^4^ Nursing CardiacServices Wythenshawe Hospital, Manchester University NHS Foundation Trust (MFT) Manchester UK; ^5^ Department of Physiotherapy The Queen Elizabeth Hospital King's Lynn NHS Foundation Trust Kings Lynn UK; ^6^ Physiotherapy Department Cambridge University Hospital NHS Foundation Trust Cambridge UK

**Keywords:** exercise interventions, multimorbidity, older adults, research waste

## Abstract

**Background and Aims:**

Multicomponent exercise interventions are recommended for older adults and for those with chronic diseases. While multiple programs have been tested, no one has yet explored how these programs were developed. This review set out to determine what development steps multicomponent exercise intervention studies that include older adults with multimorbidity have taken.

**Methods:**

Systematic review and narrative synthesis.

**Results:**

One hundred and thirty‐eight studies meeting review criteria (*Population*: adults ≥60 years with multimorbidity; *Intervention*: exercise interventions with ≥2 components; *Comparator*: any considered; *Outcome*: any considered) were retrieved. Most studies (70%) do not report intervention development actions as suggested by available guidance. Notable deviations from recommendations include limited performance of systematic review of previously published evidence, lack of engagement with theory, and few examples of design then refine.

**Conclusions:**

Exercise interventions for older adults with multimorbidity do not appear to follow best practice in terms of their developing. Disregard of development recommendations risks contributing to research redundancy and/or avoidable waste, as important steps that make sure the intervention is warranted, suitable for the population in question, and tested using optimal methods and outcome measures are overlooked.

## Key points

- Most exercise interventions do not appear to be developed in line with current recommendations.
- This risks contributing to redundant research and research waste.
- Researchers should endeavor to publish developmental work to accelerate future research.

## 1 INTRODUCTION

Multimorbidity, frequently defined as the coexistence of two or more chronic conditions in an individual,^1^ is estimated to affect ∼40% of the worlds population.^2^ Across regions, Europe has the third highest prevalence^2^; and rates are set to increase as life expectancy improves and populations age.^3^ The impact of multimorbidity cannot be understated, it is associated with significant healthcare use^3^ and therefore utilization costs.^4^ At the individual level, it results in feelings of vulnerability, loss of agency, and isolation^5^, ^6^; which can severely impact on quality of life.^7^ When asked, people with multimorbidity stated their priorities were to prevent social isolation and promote independence.^8^ To address these priorities, they suggested research should focus on the role of exercise therapy and specifically on establishing efficacy, acceptability, and its effects on important outcomes like isolation, and physical and emotional well‐being.

To date, there have been no reviews that have assessed what development steps studies testing exercise interventions in multimorbid older adult populations have taken (i.e., no assessment of how researchers have made important design decisions in relation to their intervention components and the intervention delivery). This review will address this gap in the evidence base, by evaluating study development against expert‐derived guidance on complex intervention development.

## 2 RATIONALE AND OBJECTIVES

The range of impacts of multimorbidity, and the priorities set by those who experience it, necessitate the design and testing of complex exercise interventions. In this review, we sought to explore what steps had been taken to develop exercise interventions in studies that involved older adults with multimorbidity and to assess these against current recommendations on the development of complex interventions.^9^

## 3 METHODS

We used the Cochrane Handbook for Systematic Reviews of Interventions^10^ as a guide for the review conduct and referred to the synthesis without meta‐analysis (SWiM) recommendations as an extension to the review conduct.^11^ In line with best practice, we have followed Preferred Reporting Items for Systematic Reviews and Meta‐Analyses (PRISMA) standards for reporting. Ethical approval was not necessary for the conduct of this review.

### 3.1 Protocol and registrations

The review was deposited in the International Prospective Register of Systematic Reviews (ID: CRD42020209672).

### 3.2 Eligibility criteria

We included controlled, experimental, quasi‐experimental, and pre‐test post‐test studies published in the English language up to November 2020. PICO (Population, Intervention, Comparator, Outcome) criteria are outlined below:

#### 3.2.1 Population

Studies had to include older adults (≥60 years)^12^ with multimorbidity (≥2 multiple health conditions).^1^, ^13^ We used the definition from The United Nations^12^ to define an older adult (i.e., those ≥60 years of age). In studies that targeted older adults and/or one specific condition, we considered multimorbidity to be present if mean comorbidities or mean disease counts were greater ≥2, the Charlson Comorbidity Index score was ≥2, baseline characteristics suggested the sample was multimorbid (e.g., greater than 50% of the sample had 2 or more concurrent conditions).

#### 3.2.2 Intervention

Any multicomponent exercise programs (i.e., involved ≥2 types of exercises) were included.^14^

#### 3.2.3 Comparison

Any comparisons were considered, including no comparison group, or repeated measure studies.

#### 3.2.4 Outcomes

All studies and linked reports were assessed against the Framework of Actions for Intervention Development^9^ which defines eleven key aspects of complex intervention development. Supporting Information S1: Table 1 described the actions, items considered as part of the action, and a description of the assessment process.

### 3.3 Information sources and search

Six bibliographic databases were searched from inception to November 2020 (MEDLINE [via Ovid], Embase [Via OVID], EMCARE [via Ovid], CINAHL [via EBSCO], Cochrane Library, Web of Science). Details of the exact search methods can be found in Supporting Information S1: Table 2.

### 3.4 Study selection

The screening was performed independently in duplicate via the web‐based platform Rayyan.^15^ All full texts that passed initial screening were reviewed by two members of the team; disagreements were resolved by another. All reports related to a study were collated to enable assessment of the development process.^16^

### 3.5 Data collection process and data items

Data items charted included administrative, development assessment, sample details, intervention details, and outcomes.

### 3.6 Risk of bias in individual studies

Randomized controlled trials (RCTs) were assessed against the Cochrane Collaboration's tool for assessing the risk of bias.^17^ Non‐randomized studies were assessed using the Risk Of Bias In Non‐randomized Studies of Interventions (ROBINS‐I) tool.^18^ The “mcguinlu/robvis” package was used to generate the risk of bias plots.^19^

## 4 SYNTHESIS OF RESULTS

In line with best practice, SWiM items^11^ have been used to guide the synthesis process and description of the synthesis.

### 4.1 Grouping, standardization, synthesis methods, certainty

Studies were grouped based on the number and type of exercise components they included and assessed against the 11 consensus‐driven complex intervention development actions devised by O'Cathain et al.^9^ Studies were also grouped on the basis of the “strength” of their pre‐intervention development action whereby a score of ≥3 was considered to represent more robust intervention development processes. We hypothesized that enhanced intervention development processes would be reflected in the number of exercise components as preintervention work would highlight the breadth of impairment in multimorbidity and the need for diverse options to enable participation.

Statistical exploration of the impact on outcome of the number and type of exercise components and the extent of intervention development will be presented in the planned follow‐up meta‐analysis. Heterogeneity and certainty of evidence will also be considered in this subsequent report.

## 5 RESULTS

### 5.1 Study selection

**Figure 1:**
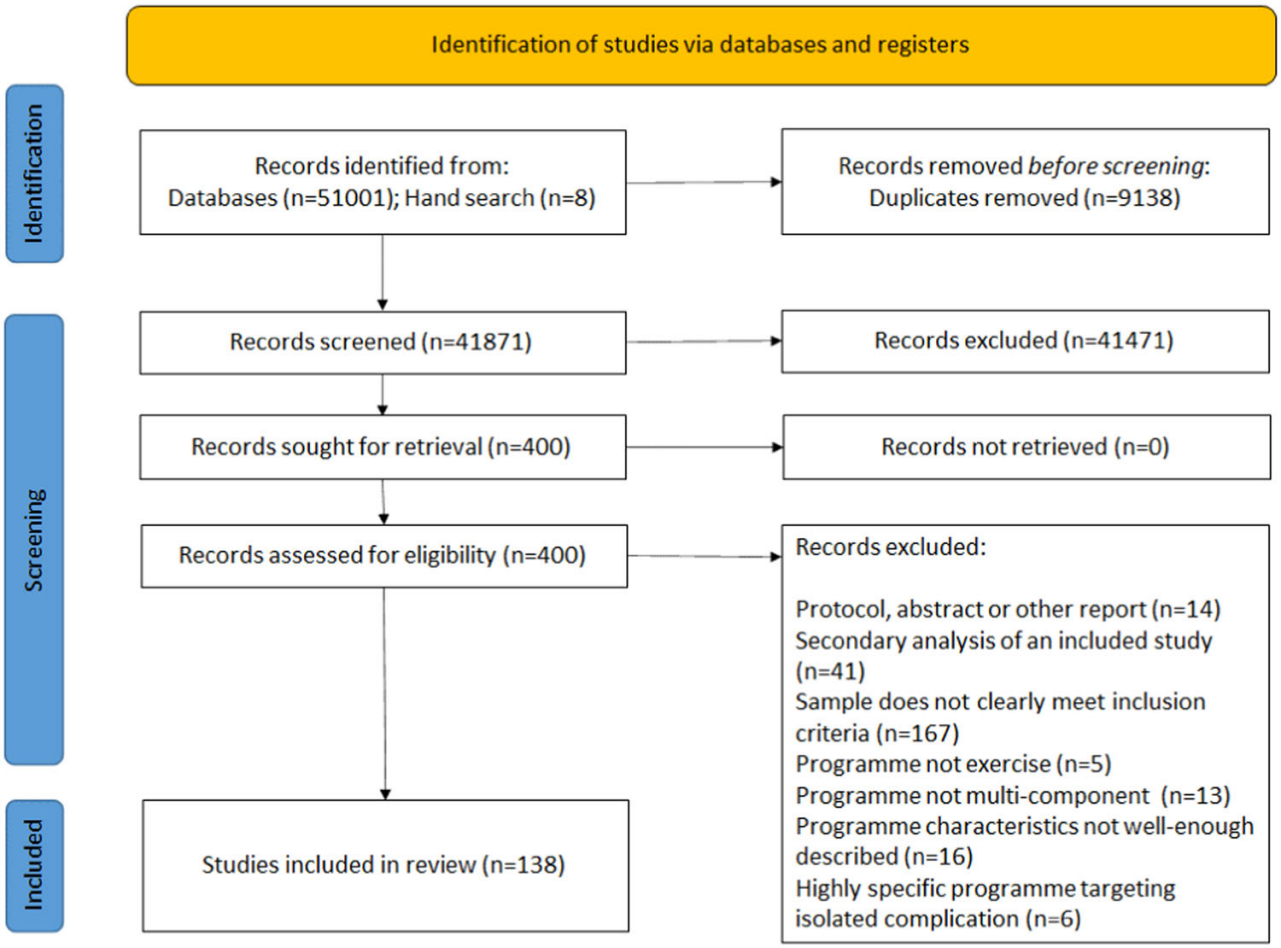
Preferred Reporting Items for Systematic Reviews and Meta‐Analyses flow diagram.

A total of 51,001 papers were retrieved. From these, 138 articles reporting unique interventions were included. We performed extensive searches to retrieve associated texts (e.g., reports of protocols, pilots, and development work) to assess predevelopment/additional outcomes resulting in a total of 259 articles. The study flow is represented in Figure 1.

### 5.2 Study characteristics

#### 5.2.1 General characteristics

Study characteristics are summarized in Supporting Information S1: Tables 3–6. Due to the volume of studies, the complete list of references is also located in the Supporting Information. The earliest report retrieved was from 1988 and the latest was from 2020. Most came from Europe (42.7%), however North America (32.6%) and Asia (10.1%) also featured strongly. Most used an RCT design (*n* = 104, 75.4%); the remaining were quasi‐experimental (*n* = 34, 24.6%).

#### 5.2.2 Participant characteristics

In total, 22,610 participants were included. The mean age was 72.1 years (range 60–92), and the mean number of comorbidities was 3.4 (range: 2–6.6); although this was poorly reported. Seven of 12 studies using a measure of morbidity used the Charlson Comorbidity Index (CCI)^20^; the mean CCI was 3.39.

Although all studies included multimorbid subjects, only four specifically stated their focus was multimorbidity. The target for most studies was a single‐chronic disease (*n* = 79; 57.2%); a lesser proportion targeted combined chronic disease (2—diseases *n* = 8; 5.8%; 3—diseases *n* = 2; 1.4%). Other popular targets included activity (*n* = 20; 14.5%) and risk factors (*n* = 19; 13.8%). Of the chronic diseases, cardiovascular diseases were represented the most (*n* = 24; 17.4%). Less represented were respiratory conditions (*n* = 14; 10.1%), cerebrovascular conditions (*n* = 11; 8%), or metabolic conditions (*n* = 9; 6.5%). Age‐related targets, that is, physical function or geriatric conditions were also represented (function *n* = 12; 8.7%; geriatric conditions *n* = 10; 7.2%; frailty *n* = 10, 7.2%).

#### 5.2.3 Intervention development assessment

**Figure 2:**
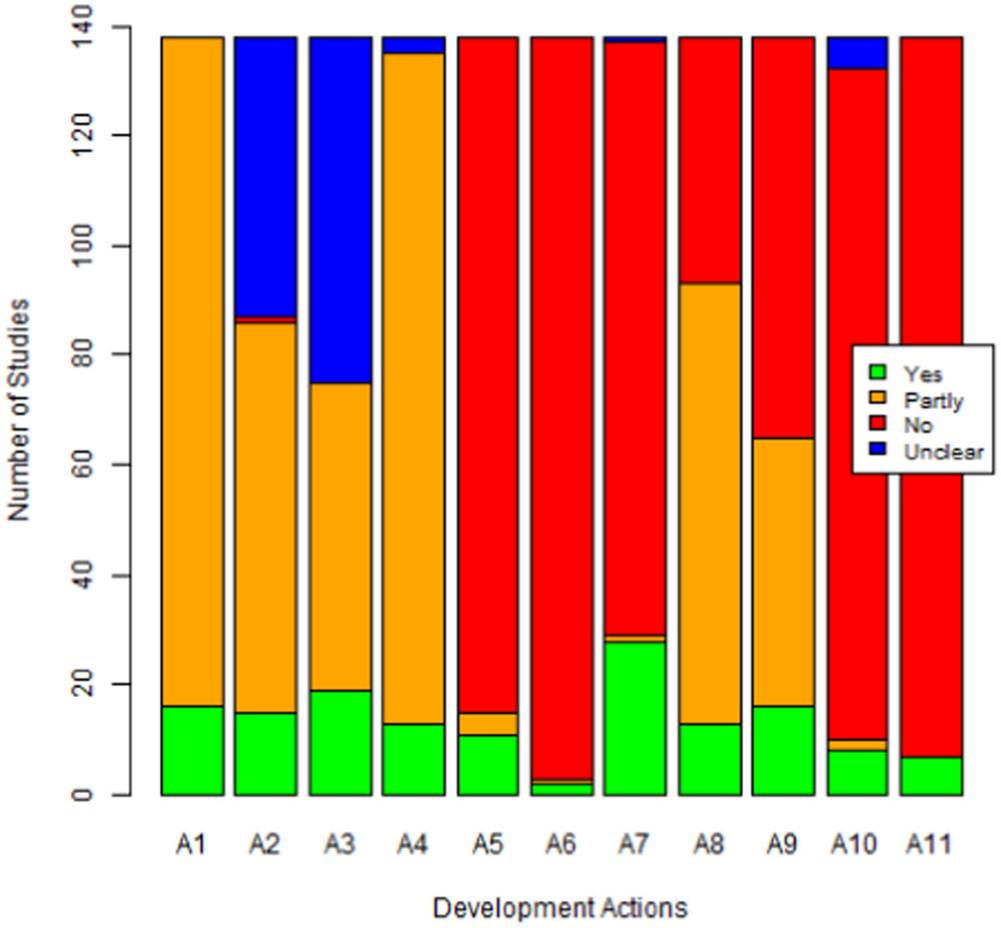
Overview of intervention development assessment.

An overview of the intervention development assessment results is presented in Figure 2. All studies were perceived to *plan the process* (A1) in some way. Fewer studies appeared to *involve stakeholders* (A2). More studies presented evidence suggesting they did *bring together a team together* (A3). The majority of studies did perform a *review of published evidence* (A4) in some way.

**Figure 3:**
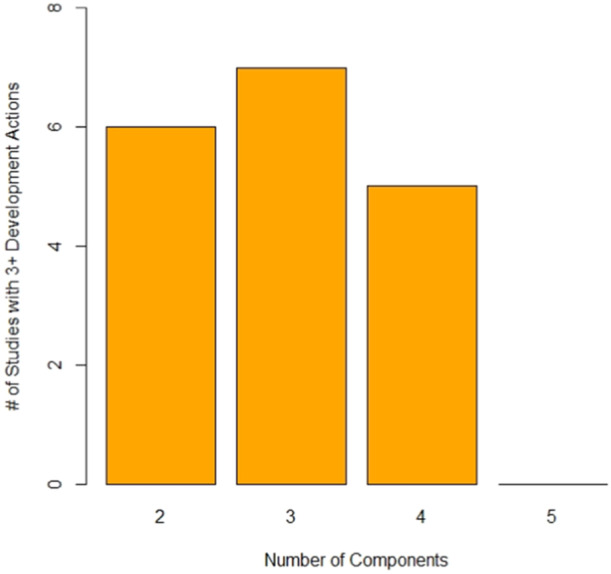
Adherence to ≥3 development criteria and number of components included in the intervention.

However, studies did not explicitly *draw on existing theories* (A5) nor *articulate program theory* (A6). Some studies did *undertake primary data collection* (A7), however most did not. Within publications, it did seem that studies had taken some steps to *understand context* (A8), however fewer presented any evidence to suggest actions to *attend to future implementation* (A9). There was very little evidence to support the concept of *design and refine* (A10) and an equally high proportion did not *end the development phase* (A11) in a manner consistent with the guidance. We defined more robust development as “meeting ≥3 development actions”; there was no relationship between a number of exercise components and the robustness of intervention development (Figure 3).

**Figure 4:**
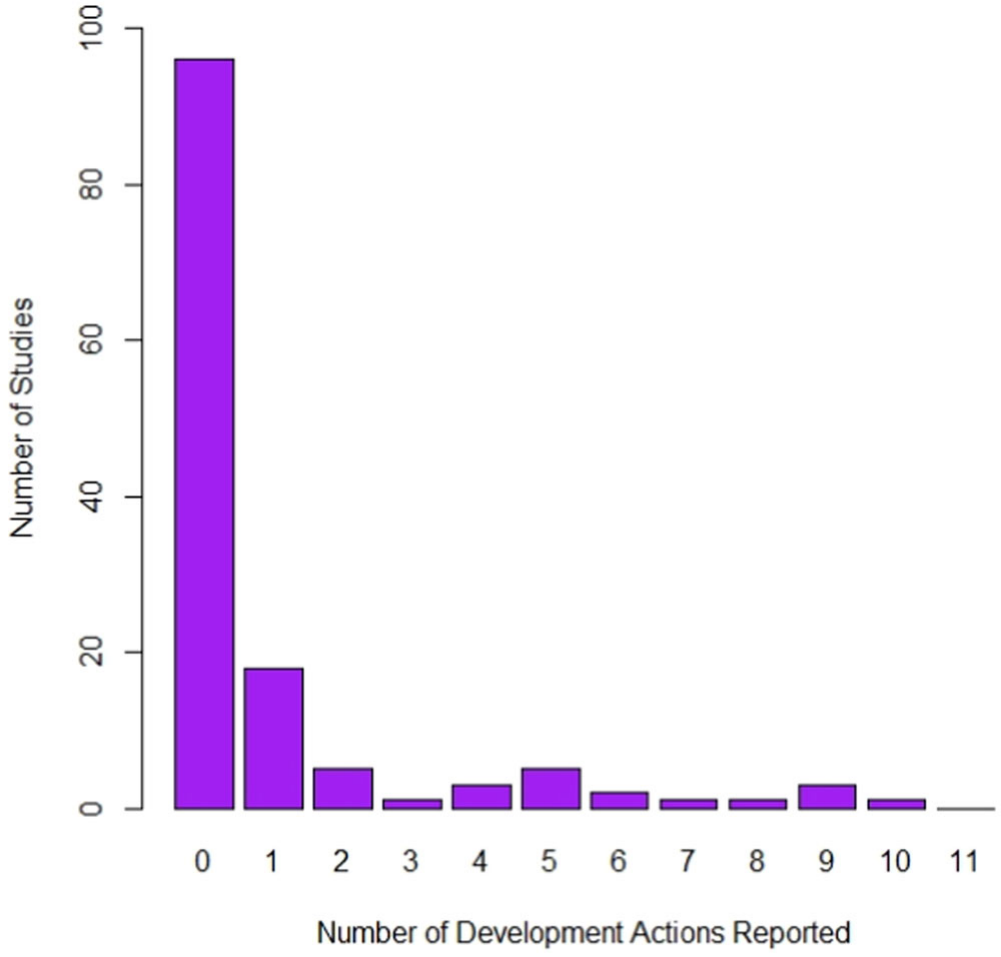
Summary of the number of development actions reported.

Overall, very few studies provided robust evidence of considering and/or acting upon these important steps when developing their intervention (Figure 4). Most studies (70%) provided no descriptions of actions that were compatible with the complex intervention development framework. Only 19 studies (13.7%) were graded as “yes provided evidence” in three or more developmental action categories. Of these studies, the mean achieved was 5.0 (range: 3–9). Within these studies, planning the process (79.9%), bringing together a team (84.2%), and attending to the future implementation (78.9%) were the most frequently described actions.

### 5.3 Risk of bias within studies

The risk of bias was assessed using the ROBINS‐I^18^ and Cochrane Risk of Bias 2 tools (see Supporting Information S1: Figures 1 and 2).^10^ Robustness of intervention development did not necessarily translate to methodologically superior studies. Of the 19 developmentally better studies, none were assessed to be of low risk of bias, 16 were graded as presenting “some concerns” (84.2%) and 3 were judged to be at high risk of bias (15.8%).

## 6 DISCUSSION

### 6.1 General interpretation

This review found that studies did not frequently report pre‐intervention development work. Although we expect in some cases non‐reporting of development activities rather than non‐completion of development activities explain our findings, the results remain concerning. Poor development of interventions increases the risk of testing interventions that are not effective or interventions that are not acceptable to the target population.^9^ Reporting of development activities provides more data and information for future intervention development to be built upon.

In terms of specific intervention development actions taken/not taken, there were a few standout findings. Most studies (86%) were graded as only partly achieving a “review of published evidence” based on the background and rationale presented within the publication. Very few (10.9%) provided emphatic evidence of synthesizing and critically appraising previous literature addressing the same topic via a scoping, systematic, or rapid review. Rather, reports gave the impression of being selective of the studies used to justify their research. This confirmation or optimism bias, especially in the context of a null result, raises doubt regarding the original justification of the research. Authorities on avoidable waste in research have called for the mandating of systematic reviews of existing evidence as part of research funding agreements.^21^

Only two studies articulated their program theory (a narrative, often diagrammatical, that conveys how an intervention will lead to an outcome) and 12 drew on an existing theory. Evidence that theory use (whether existing or novel) delivers better outcomes in health behavior change interventions is generally lacking.^22^ However, there are more promising signals for efficacy in exercise trials;^22^ particularly in older adults.^23^ Regardless of their role in changing outcomes, advocates argue theory utilization helps to clarify intervention actions, implementation processes, outcome measures, logistics, and evaluation methods.^24^

Collecting informative “primary” information and “designing and refining” were further development actions that were suboptimally performed. Feasibility or pilot studies allow researchers to test the validity of the research question, design, and methods and they have been shown to reduce research waste.^25^ Equally, there is emerging evidence of the value of patient involvement in the design phase of research, particularly around relevancy and in addressing researcher assumptions early on.^26^, ^27^, ^28^

### 6.2 Application of findings in clinical practice

The impact of these results is most applicable to the research setting. Given that development guidance has been around for some time (the Medical Research Council framework for complex intervention development first appeared in 2000^29^); these findings are surprising. In terms of clinical practice, it could be extrapolated that we may not have optimal information to guide exercise prescriptions in multimorbid older adults, as most research reporting to date has not adhered to best practices.

### 6.3 Comparison with other reviews

We were able to retrieve limited reviews for comparison. A narrative review examining the impact of older adults'; involvement in physical activity intervention development, found all eligible studies (*n* = 10) reported positive relationships between involvement and outcomes like satisfaction, participation, and adherence.^30^

### 6.4 Limitations of the review process and evidence

This review was large and comprehensive and adopted robust methods that are in line with best practice recommendations. However, there are limitations that must be acknowledged. Firstly, some studies included pre‐dated intervention development guidelines, and therefore it may be unfair to judge them against contemporary standards. Second, as other publications have noted, there is a limited appetite within journals to publish “messy” research such as papers that report how an intervention has been developed.^31^ Third, presence/absence conditions may unfairly penalize studies that have not, for reasons of reporting guidelines and journal requirements, been able to incorporate information relating to intervention design. Fourthly, while assessment criteria and the evidence regarded as satisfying that criteria were established, the judgment is nevertheless, subjective. Lastly, studies published after the search date would have been missed. A search re‐run conducted in January 2024 in Medline (via Ovid) identified four studies that would have met our inclusion criteria. These did not report any substantially different patterns of intervention development, so their inclusion would not have materially affected the results as presented.

## 7 CONCLUSIONS

The review has revealed that many exercise interventions tested in multimorbid populations, have not reported intervention design actions in line with current recommendations. Even with equivocal results of efficacy, a well‐reported intervention development process can accelerate future work. As such, non‐reporting of this process, even when it does not equate to poorly conceived and researched interventions, contributes to avoidable research waste. We have identified important steps in this review, such as reviewing systematic reviews or updating/conducting new reviews, that regularly appear to be overlooked. Further measures should be taken to make sure the intervention design is suitable for, and meets the needs of the population in which it is going to be tested.

## AUTHOR CONTRIBUTIONS

*Conception and design, acquisition of data, analysis and interpretation of data, drafted the article, revised the draft critically for important intellectual content, gave final approval of the version to be published, agreed to be accountable for all aspects of the work*: Faye Forsyth. *Acquisition of data, revised the draft critically for important intellectual content, gave final approval of the version to be published, agreed to be accountable for all aspects of the work*: Chien Lin Soh. *Acquisition of data, revised the draft critically for important intellectual content, gave final approval of the version to be published, agreed to be accountable for all aspects of the work*: Natasha Elks. *Acquisition of data, revised the draft critically for important intellectual content, gave final approval of the version to be published, agreed to be accountable for all aspects of the work*: Helen Lin. *Acquisition of data, revised the draft critically for important intellectual content, gave final approval of the version to be published, and agreed to be accountable for all aspects of the work*: Kris Bailey. *Acquisition of data, revised the draft critically for important intellectual content, gave final approval of the version to be published, agreed to be accountable for all aspects of the work*: Rosalie Brooman‐White. *Analysis and interpretation of data, revised the draft critically for important intellectual content, gave final approval of the version to be published, agreed to be accountable for all aspects of the work*: Scott Rowbotham. *Analysis and interpretation of data, revised the draft critically for important intellectual content, gave final approval of the version to be published, and agreed to be accountable for all aspects of the work*: Jonathan Mant. *Acquisition of data, analysis and interpretation of data, revised the draft critically for important intellectual content, gave final approval of the version to be published, agreed to be accountable for all aspects of the work*: Peter Hartle. *Conception and design, acquisition of data, analysis and interpretation of data, revised the draft critically for important intellectual content, gave final approval of the version to be published, agreed to be accountable for all aspects of the work*: Christi Deaton. All authors have read and approved the final version of the manuscript.

## CONFLICT OF INTEREST STATEMENT

The authors declare no conflict of interest.

## TRANSPARENCY STATEMENT

The lead author Faye Forsyth affirms that this manuscript is an honest, accurate, and transparent account of the study being reported; that no important aspects of the study have been omitted; and that any discrepancies from the study as planned (and, if relevant, registered) have been explained.

## Supporting information

Supporting information.

## Data Availability

Data will be made available upon request. Faye Forsyth (corresponding author) had full access to all of the data in this study and takes complete responsibility for the integrity of the data and the accuracy of the data analysis.
