# Supplementary material for: Development steps of multimodal exercise interventions for older adults with multimorbidity: A systematic review
Source: Health Sci Rep. 2024 Jul 1;7(7):e2190. doi: 10.1002/hsr2.2190 (PMC11215533; doi:10.1002/hsr2.2190)
Supplement: Supplementary file 1 — Supporting information. [file HSR2-7-e2190-s001.docx]

**Supplementary Material**

**Contents**

[Supplementary Table 1: Complex intervention development actions, action criteria and description of assessment process 2](#_Toc149294213)

[Supplementary Table 2: Search strategies 6](#_Toc149294214)

[Supplementary Table 3: Two component studies 12](#_Toc149294215)

[Supplementary Table 4: Three component studies 18](#_Toc149294216)

[Supplementary Table 5: Four component studies 23](#_Toc149294217)

[Supplementary Table 6: Five component studies 27](#_Toc149294218)

[Supplementary Figure 1: ROBINS-I 28](#_Toc149294219)

[Supplementary Figure 2: Cochrane Risk of Bias 2 Plot 29](#_Toc149294220)

[References 33](#_Toc149294221)

# Supplementary Table 1: Complex intervention development actions, action criteria and description of assessment process

| **Action** | **Potential criteria to consider as part of the action** | **Description of method of searching for action items within primary study and/or associated publications included in the review*** |
| --- | --- | --- |
| A1: Plan the process | Identify the problem to be targeted and refine understanding of it throughout. | Evidence presented in introduction, background, rationale, protocol. |
|  | Assess whether the problem is a priority. | Evidence of consulting or involving the end user(s) e.g., deliverers and users. |
|  | Consider which aspects of the problem are amendable to change. | Evidence of consulting or involving the end user(s) e.g., deliverers and users. |
|  | Ask whether a new intervention is really needed and if the potential benefit of the new intervention justifies the cost of the development. | Evidence presented in introduction, background, rationale, protocol.  Evidence of cost/benefit analysis or other considerations of need or cost (before or after). |
|  | Determine the time needed to undertake intervention development. | Evidence of associated publication detailing the intervention development and implementation process. |
|  | Obtain sufficient resource/funding for the intervention development study | Statements of funding. |
|  | Draw on one or more of the many published intervention development approaches, recognizing that the is no evidence about which approach is best and apply flexibly depending on the problem and context. | Reference to an established or bespoke intervention development approach. |
|  | Involve stakeholders during the planning process. | Evidence of consulting or involving the end user(s) e.g., deliverers and users. |
|  | Produce a protocol detailing the processes to be undertaken to develop the intervention. | Evidence of associated publication detailing the intervention development and implementation process. |
| A2: Involve stakeholders | Work closely with relevant stakeholders throughout the development process: patients, the public, the target population, service providers, those who pay for health and social services or interventions, policymakers and intervention design specialists. | Evidence of consulting or involving the end user(s) e.g., deliverers and users. |
|  | Develop a plan at the start of the process to integrate public and patient involvement into the intervention development process. | Evidence of end user involvement, including assessment of acknowledgements and PPI statements. |
|  | Identify the best way of working with each type of stakeholder, from consultation through to coproduction, acknowledging that different ways may be relevant for different stakeholders at different times. | Evidence of consulting or involving the end user(s) e.g., deliverers and users.  Evidence of end user involvement, including assessment of acknowledgements and PPI statements. |
|  | Use creative activities within the team meetings wo work with stakeholders to understand the problem and generate ideas for the intervention | Evidence of consulting or involving the end user(s) e.g., deliverers and users.  Evidence of end user involvement, including assessment of acknowledgements and PPI statements. |
| A3: Bring together a team | Include within the development team individuals with relevant expertise: in the problem to be addressed by the intervention inclusion those with personal experience of the problem, in behavior change when the intervention aims to change behavior, in maximizing engagement of stakeholders and with a strong track record in designing complex interventions. | Evidence of roles detailed within methods and/or other sections.  Examination of author list and acknowledgements. |
|  | It may be hard to make final decisions about the content, format and delivery of the intervention, so only some team members may do this. There is no consensus about the size or constituency of the team that makes these final decisions, but it is important early on to agree a process for making decision within the team. | Evidence of roles detailed within methods and/or other sections of the publication e.g., contributions.  Examination of author list and acknowledgements. |
| A4: Review published research evidence | Review published research evidence before starting to develop the intervention and throughout the development process for example, to identify existing interventions, to understand the evidence base for each proposed substantive intervention component. | Evidence presented in introduction, background, rationale, protocol. |
|  | Look for and take into account, evidence that the proposed intervention may not work in the way intended. | Evidence presented in introduction, background, rationale, protocol. |
| A5: Draw on existing theory | Identify an existing theory or framework of theories to inform the intervention at the start of the process, for example, behavior change or implementation. | Reference to an established or bespoke theory or framework of theories. |
|  | Where relevant, draw on more than one existing theory or framework for example, both psychological and organisational theories. | Reference to an established or bespoke theory or framework of theories |
| A6: Articulate programme theory | Develop a programme theory. The programme theory may draw on existing theories. Aspects of the programme theory can be represented by a logic model or a set of models. | Evidence of a programme theory presented in introduction, background, rationale, protocol. |
|  | Test and refine the programme theory throughout the development process. | Evidence of a programme theory presented in introduction, background, rationale, protocol. |
| A7: Undertake primary data collection | Use of a wide range of research methods throughout, for example, qualitative research to understand the context in which the intervention will operate, quantitative methods to measure change in intermediate outcomes. | Evidence of associated ‘data generating’ publications. |
| A8: Understand context | Understand the context in which the intervention will be implemented. Context may include population, individuals, physical location or geographical setting; social, economic, cultural and political influences and factors affecting implementation, for example, organization, funding and policy. | Evidence of consideration of context presented in introduction, background, rationale, protocol, or other associated papers. |
| A9: Pay attention to future implementation | From the start, understand facilitators and barriers to reaching the relevant population, future use of the intervention, ‘scale up’ and sustainability in real world contexts. | Evidence of consideration of the ‘real world’ presented in introduction, background, rationale, protocol, or other associated papers. |
| A10: Design and refine | Generate ideas about content, format and delivery with stakeholders. | Evidence of consulting or involving the end user(s) e.g., deliverers and users.  Evidence of end user involvement, including assessment of acknowledgements and PPI statements. |
|  | Once an early version or prototype of the intervention is available, refine or optime it using a series of iterations. Each iteration includes assessment of how acceptable, feasible and engaging the intervention is, including potential harms and unintended consequences, resulting in refinements to the intervention, Repeat the process until uncertainties are resolved. | Evidence of pilot, feasibility, quasi-experimental pre-test-post-test studies to evaluate changes in or efficacy of outcomes, naturalistic prospective studies, design-based experiments, mixed method evaluations, process evaluations. |
|  | Check that the proposed mechanisms of action are supported by early testing. | Evidence of pilot, feasibility, quasi-experimental pre-test-post-test studies to evaluate changes in or efficacy of outcomes, design-based experiments, mixed method evaluations, process evaluations. |
| A11: End the development phase | Describe the intervention, write up intervention development process. | Evidence of associated publications detailing the intervention development and implementation process. |
| *If all or most of the items considered as part of the action were demonstrated, the action was marked as **‘YES’**. If some of the items to consider as part of the action were demonstrated, the action was marked as **‘PARTLY’**. If none of the items to consider as part of the action were demonstrated, the action was marked as **‘NO’**. If there was reference to an action that may or may not be related to development, the action was marked an **‘UNCLEAR’** (for example, if multiple professionals were listed as part of the team but not referred to specifically within text relating to developing an intervention, this was marked as unclear). | | |

# Supplementary Table 2: Search strategies

| EMCARE  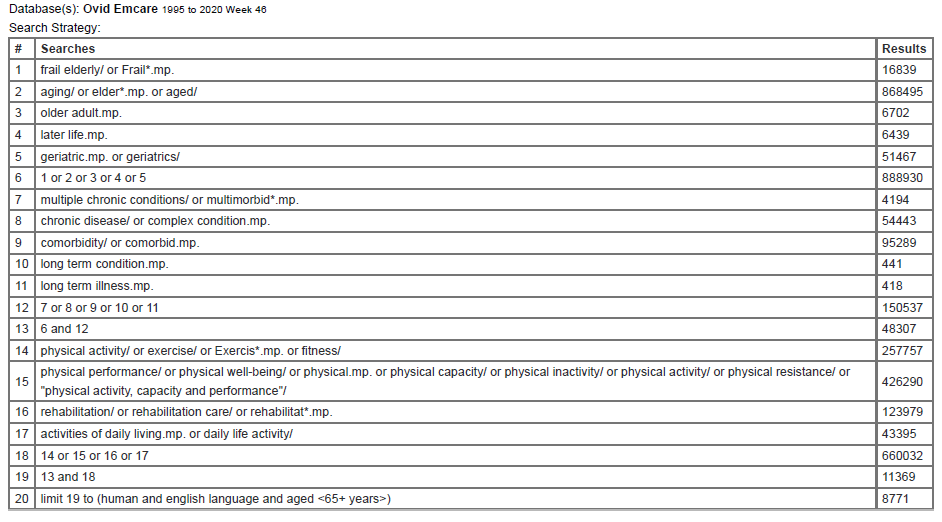 |
| --- |
| Web of Science  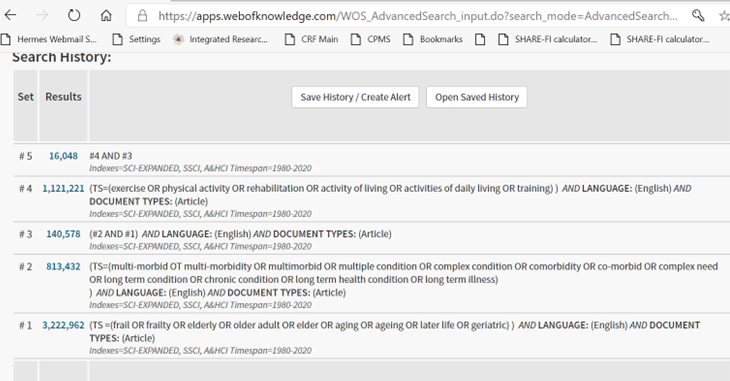 |
| CINAHL  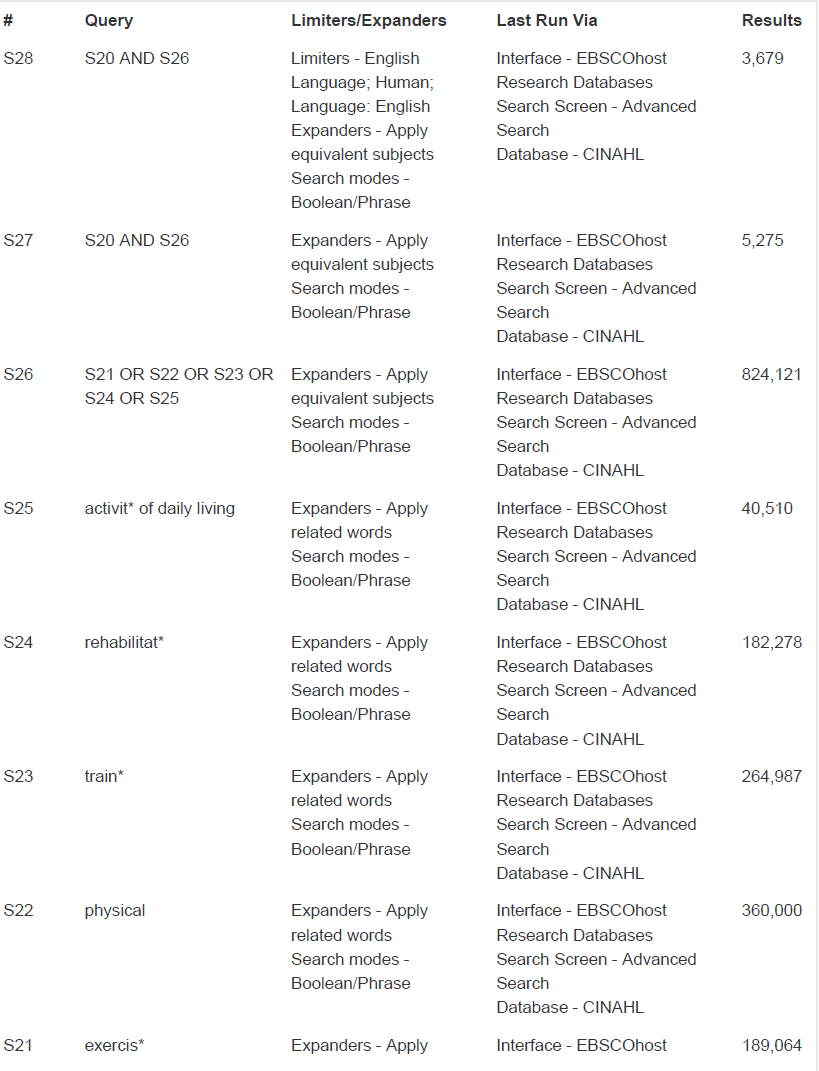 |
|  |
| AMED  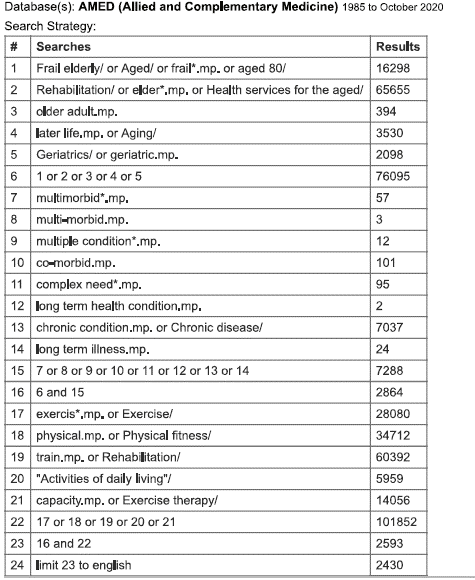 |
| EMBASE  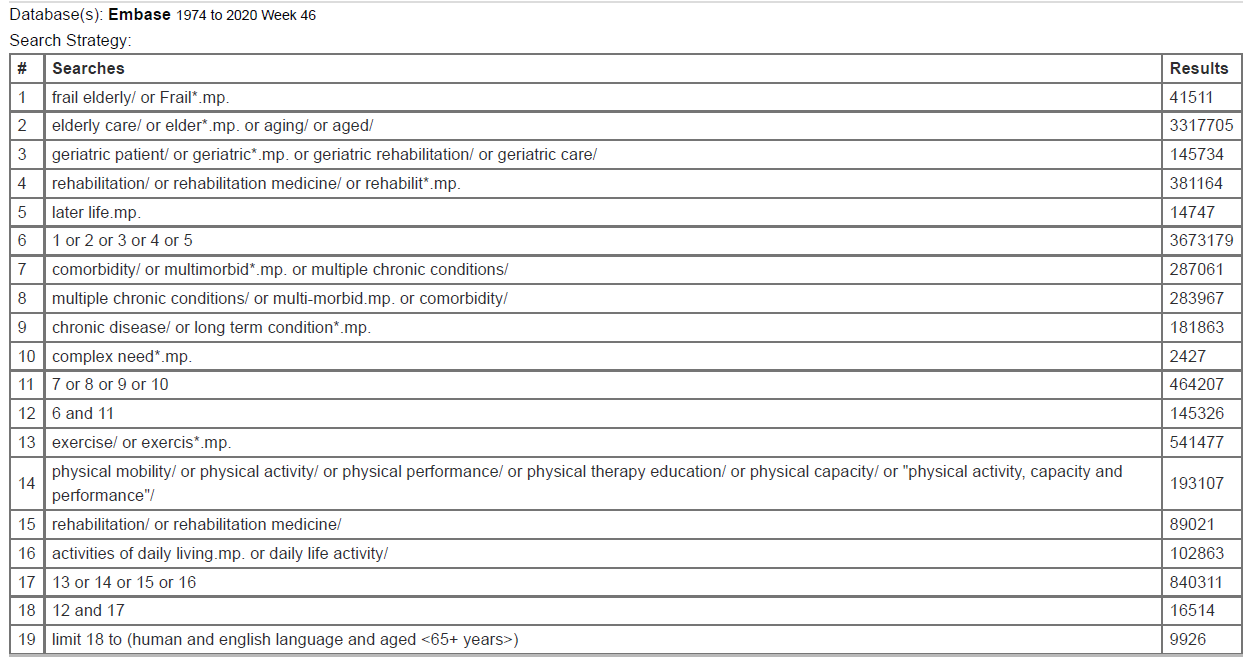 |
| MEDLINE  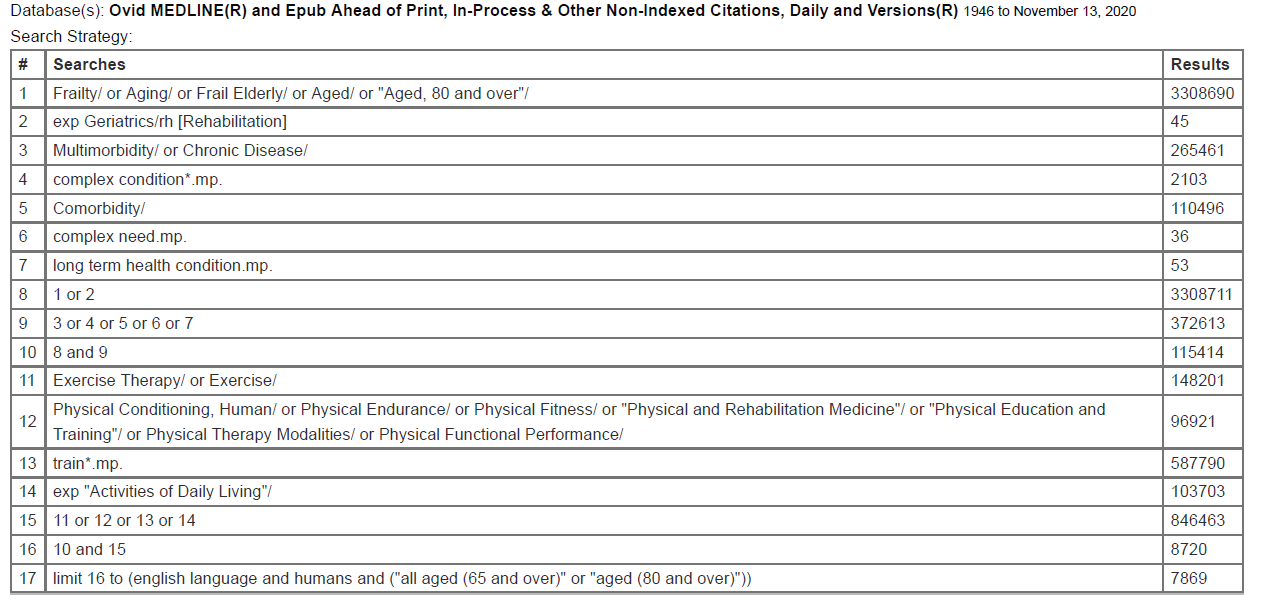 |
| COCHRANE  Search Name: Exercise Systematic Review  Date Run: 16/11/2020 16:44:10  Comment: Cochrane Library search 166 Nov 2020  ID Search Hits  #1 ("Frail*"):ti,ab,kw (Word variations have been searched) 2459  #2 ("elder*"):ti,ab,kw (Word variations have been searched) 95390  #3 ("aged"):ti,ab,kw (Word variations have been searched) 696422  #4 ("older adult"):ti,ab,kw (Word variations have been searched) 13112  #5 ("aging"):ti,ab,kw (Word variations have been searched) 696418  #6 ("ageing"):ti,ab,kw (Word variations have been searched) 696418  #7 ("later life"):ti,ab,kw (Word variations have been searched) 463  #8 ("geriatric"):ti,ab,kw (Word variations have been searched) 8504  #9 #1 or #2 or #3 or #4 or #5 or #6 or #7 or #8 714999  #10 ("multi-morbid*"):ti,ab,kw (Word variations have been searched) 93  #11 ("multimorbid*"):ti,ab,kw (Word variations have been searched) 413  #12 ("multiple condition*"):ti,ab,kw (Word variations have been searched) 56  #13 ("complex condition*"):ti,ab,kw (Word variations have been searched) 110  #14 ("co-morbid*"):ti,ab,kw (Word variations have been searched) 4040  #15 ("comorbid*"):ti,ab,kw (Word variations have been searched) 20743  #16 ("complex need*"):ti,ab,kw (Word variations have been searched) 92  #17 ("long term condition*"):ti,ab,kw (Word variations have been searched) 219  #18 ("long-term condition*"):ti,ab,kw (Word variations have been searched) 219  #19 ("chronic condition*"):ti,ab,kw (Word variations have been searched) 1920  #20 ("long term health condition*"):ti,ab,kw (Word variations have been searched) 28  #21 ("long term illness*"):ti,ab,kw (Word variations have been searched) 55  #22 #10 or #11 or #12 or #13 or #14 or #15 or #16 or 17 or #18 or #19 or #20 or #21 206987  #23 ("exercis*"):ti,ab,kw (Word variations have been searched) 26  #24 ("physical"):ti,ab,kw (Word variations have been searched) 118018  #25 ("train*"):ti,ab,kw (Word variations have been searched) 105200  #26 ("rehabilitat*"):ti,ab,kw (Word variations have been searched) 2  #27 ("activities of daily living"):ti,ab,kw (Word variations have been searched) 10960  #28 #23 or #24 or #25 or #26 or #27 202750  #29 #9 and #22 and #28 17560  #30 #29 in Trials, Clinical Answers 16865 |

# Supplementary Table 3: Two component studies

| **AUTHORS &**  **ACRONYM** | **YEAR &**  **COUNTRY** | **DESIGN &**  **TYPE** | **SAMPLE SIZE**  **(n)** | **MEAN AGE**  **(YEARS)** | **SPECIFIC DISEASE TARGETED** | **COMORBIDITIES**  **(MEAN)** | **COMORBIDITY MEASURE (SCORE & NAME)** | **BASELINE PERCENTAGES OF CONCURRENT CHRONIC CONDITIONS SUGGEST MM** | Plan the process | Involve Stakeholders | Bring together a team | Review published evidence | Draw on existing theories | Articulate programme theory | Undertake primary data collection | Understand context | Attend to future implementation | Design and refine | End the development phase |
| --- | --- | --- | --- | --- | --- | --- | --- | --- | --- | --- | --- | --- | --- | --- | --- | --- | --- | --- | --- |
| Bourne  et al.^1^ | 2017 | RCT | 90 | 69.7 | COPD | NS | NS | ✓ | Y | Y | Y | Y | N | N | UC | Y | Y | UC | N |
| My-PR | UK | PARALLEL |  |  |  |  |  |  |  |  |  |  |  |  |  |  |  |  |  |
| Budui  et al.^2^ | 2019 | QUASI-EX | 259 | 69.3 | OBESITY | NS | NS | ✓ | P | UC | UC | P | N | N | Y | P | N | N | N |
| No acronym | ITALY | PRE-TEST-POST-TEST |  |  |  |  |  |  |  |  |  |  |  |  |  |  |  |  |  |
| Dimori  et al.^3^ | 2018 | QUASI-EX | 39 | 86.5 | SARCO | NS | 4.7  CIRS | NA | P | UC | UC | P | N | N | N | P | N | N | N |
| No acronym | ITALY | PRE-TEST-POST-TEST |  |  |  |  |  |  |  |  |  |  |  |  |  |  |  |  |  |
| Hassan  et al. ^4^ | 2016 | QUASI-EX | 40 | 60.3 | COPD | NS | NS | ✓ | P | U | U | P | N | N | N | P | N | N | N |
| No acronym | EGYPT | PRE-TEST-POST-TEST |  |  |  |  |  |  |  |  |  |  |  |  |  |  |  |  |  |
| Higashimoto et al. ^5^ | 2016 | QUASI-EX | 70 | 71.8 | COPD | NS | 2.3 CCI | NA | P | UC | UC | P | N | N | Y | P | N | N | N |
| No acronym | JAPAN | PRE-TEST-POST-TEST |  |  |  |  |  |  |  |  |  |  |  |  |  |  |  |  |  |
| Marzolini  et al. ^6^ | 2016 | QUASI-EX | 53 | 67.5 | TIA | NS | NS | ✓ | P | P | P | P | N | N | N | N | N | N | N |
| No acronym | CANADA | PRE-TEST-POST-TEST |  |  |  |  |  |  |  |  |  |  |  |  |  |  |  |  |  |
| Allen  et al. ^7^ | 2018 | RCT | 107 | 76 | REDUCED FUNCTION | NS | NS | ✓ | P | P | UC | P | N | N | N | P | P | N | N |
| PRIME & Fit for Life trial | USA | PARALLE |  |  |  |  |  |  |  |  |  |  |  |  |  |  |  |  |  |
| Chen  et al. ^8^ | 2016 | RCT | 127 | 79.4 | WHEEL CHAIR USER | 2.1 | NS | NA | Y | Y | Y | P | N | N | Y | Y | Y | Y | Y |
| WSEB | TAIWAN | CLUSTER |  |  |  |  |  |  |  |  |  |  |  |  |  |  |  |  |  |
| Gine-Garriga et al. ^9^ | 2009 | RCT | 362 | 67.7 | SEDENT | NS | NS | ✓ | P | UC | P | P | P | N | Y | P | P | N | N |
| PPAF | SPAIN | PARALELL |  |  |  |  |  |  |  |  |  |  |  |  |  |  |  |  |  |
| Hellberg,  et al. ^10^ | 2018 | RCT | 151 | 66 | CKD | NS | NS | ✓ | p | UC | UC | P | N | N | N | N | N | N | N |
| RENEXC-A | SWEDEN | PARALLEL |  |  |  |  |  |  |  |  |  |  |  |  |  |  |  |  |  |
| Hoffman et al. ^11^ | 2014 | QUASI-EX | 7 | 64.6 | CANCER | 5.9 | NS | NA | Y | Y | Y | P | Y | N | Y | Y | Y | Y | N |
| No acronym | USA | PRE-TEST-POST-TEST |  |  |  |  |  |  |  |  |  |  |  |  |  |  |  |  |  |
| Johansen  et al. ^12^ | 2012 | QUASI-EX | 302 | 80.5 | REHAB REFERRAL | NS | NS | ✓ | P | P | P | P | N | N | P | N | N | N | N |
| No acronym | NORWAY | PRE-TEST-POST-TEST |  |  |  |  |  |  |  |  |  |  |  |  |  |  |  |  |  |
| Peiris  et al. ^13^ | 2018 | QUASI-EX | 58 | 60 | MET SYN | 3.2 | NS | NA | P | P | P | P | P | N | N | P | N | N | N |
| TAKE ACTION NOW | AUSTRALIA | PRE-TEST-POST-TEST |  |  |  |  |  |  |  |  |  |  |  |  |  |  |  |  |  |
| Sajatovic  et al. ^14^ | 2017 | RCT | 30 | 70 | PD | NS | 2.6 CCI | NA | P | P | UC | P | N | N | N | P | N | N | N |
| EXCEED | USA | PARALLEL |  |  |  |  |  |  |  |  |  |  |  |  |  |  |  |  |  |
| Zgibor  et al. ^15^ | 2017 | RCT | 462 | 72.7 | ARTHRITIS & MULTI-MORBIDITY | NS | NS | ✓ | Y | Y | Y | Y | Y | N | N | Y | Y | Y | Y |
| No acronym | USA | CLUSTER |  |  |  |  |  |  |  |  |  |  |  |  |  |  |  |  |  |
| Boxall  et al. ^16^ | 2005 | RCT | 46 | 76.7 | COPD | NS | NS | ✓ | P | UC | UC | P | N | N | N | P | N | N | N |
| No acronym | AUSTRALIA | PARALLEL |  |  |  |  |  |  |  |  |  |  |  |  |  |  |  |  |  |
| Morey  et al. ^17^ | 2003 | RCT | 134 | 71.9 | SEDENT | 3.8 | NS | NA | P | UC | UC | P | N | N | N | P | N | N | N |
| No acronym | USA | PARALLEL |  |  |  |  |  |  |  |  |  |  |  |  |  |  |  |  |  |
| Serra-Rexach  et al. ^18^ | 2011 | RCT | 40 | 92 | OLDEST OLD | NS | NS | ✓ | P | UC | UC | P | N | N | N | N | N | N | N |
| STRONG | SPAIN | PARALLEL |  |  |  |  |  |  |  |  |  |  |  |  |  |  |  |  |  |
| Hetherington et al. ^19^ | 2018 | RCT | 245 | 78.7 | CARE HOME RESIDENT | 5 | NS | NA | Y | Y | Y | P | N | N | N | Y | Y | UC | N |
| Muscling Up Against Disability | AUSTRALIA | STEP WEDGE |  |  |  |  |  |  |  |  |  |  |  |  |  |  |  |  |  |
| Caminiti  et al. ^20^ | 2011 | RCT | 21 | 68 | HEART FAILURE | NS | NS | ✓ | P | UC | UC | P | N | N | N | N | N | N | N |
| No acronym | ITALY | PARALLEL |  |  |  |  |  |  |  |  |  |  |  |  |  |  |  |  |  |
| Chen  et al. ^21^. | 2015 | RCT | 54 | 66 | CHRONIC ILLNESS | NS | NS | ✓ | P | P | UC | P | N | N | N | N | N | N | N |
| No acronym | TAIWAN | PARALLEL |  |  |  |  |  |  |  |  |  |  |  |  |  |  |  |  |  |
| Erbs  et al. ^22^ | 2010 | RCT | 37 | 61 | ADVANCED HEART FAILURE | NS | NS | ✓ | P | P | UC | P | N | N | N | N | N | N | N |
| No acronym | GERMANY | PARALLEL |  |  |  |  |  |  |  |  |  |  |  |  |  |  |  |  |  |
| Garcia Diaz  et al. ^23^ | 2019 | QUASI-EX | 55 | 77.3 | DIABETES | NS | NS | ✓ | P | P | UC | P | N | N | N | N | N | N | N |
| No acronym | SPAIN | PRE-TEST-POST-TEST |  |  |  |  |  |  |  |  |  |  |  |  |  |  |  |  |  |
| Gielen  et al. ^24^ | 2012 | RCT | 30 | 72 | HEART FAILURE | NS | NS | ✓ | P | UC | UC | P | N | N | N | N | N | N | N |
| LEICA | GERMANY | PARALLEL |  |  |  |  |  |  |  |  |  |  |  |  |  |  |  |  |  |
| Hegbom  et al. ^25^ | 2006 | RCT | 30 | 63.6 | AF | NS | NS | ✓ | P | P | P | P | N | N | N | N | N | N | N |
| No acronym | NORWAY | PARALLEL |  |  |  |  |  |  |  |  |  |  |  |  |  |  |  |  |  |
| Miche  et al. ^26^ | 2006 | QUASI-EX | 42 | 67.5 | HEART FAILURE & DIABETES | NS | NS | ✓ | P | P | UC | P | N | N | N | P | N | N | N |
| No acronym | GERMANY | PRE-TEST-POST-TEST |  |  |  |  |  |  |  |  |  |  |  |  |  |  |  |  |  |
| Miche  et al. ^27^ | 2003 | QUASI-EX | 75 | 65 | HEART FAILURE | NS | NS | ✓ |  |  |  |  |  |  |  |  |  |  |  |
| No acronym | GERMANY | PRE-TEST-POST-TEST |  |  |  |  |  |  |  |  |  |  |  |  |  |  |  |  |  |
| Miche  et al. ^28^ | 2008 | QUASI-EX | 285 | 67.2 | HEART FAILURE | NS | NS | ✓ | P | P | P | P | N | N | N | P | N | N | N |
| No acronym | GERMANY | PRE-TEST-POST-TEST |  |  |  |  |  |  |  |  |  |  |  |  |  |  |  |  |  |
| Padberg  et al. ^29^ | 2004 | RCT | 30 | 70 | CHRONIC VENOUS PROBLEMS | NS | NS | ✓ | P | P | UC | P | N | N | N | N | N | N | N |
| No acronym | USA | PARALLEL |  |  |  |  |  |  |  |  |  |  |  |  |  |  |  |  |  |
| Thompson  et al. ^30^ | 1988 | RCT | 35 | 73.3 | MULTI-MORBIDITY | NS | NS | ✓ | P | UC | UC | P | N | N | N | N | N | N | N |
| No acronym | USA | PARALLEL |  |  |  |  |  |  |  |  |  |  |  |  |  |  |  |  |  |
| Yu  et al. ^31^ | 2007 | RCT | 153 | 75.4 | HEART FAILURE | 2.7 | NS | NA | P | UC | UC | P | N | N | N | N | N | N | N |
| No acronym | CHINA | PARALLEL |  |  |  |  |  |  |  |  |  |  |  |  |  |  |  |  |  |
| Barker  et al. ^32^ | 2018 | RCT | 17 | 69.3 | REQUIRING REHAB | 4 | NA | NA | P | P | P | P | N | N | Y | P | P | N | N |
| No acronym | AUSTRALIA | PARALLEL |  |  |  |  |  |  |  |  |  |  |  |  |  |  |  |  |  |
| Narlon  et al. ^33^ | 2018 | RCT | 127 | 67.4 | SELF-REPORTED COGNITIVE PROBLEM | NS | NS | ✓ | P | P | P | Y | N | N | N | N | N | N | N |
| The Multiple-Modality, Mind-Motor Study | CANADA | PARALLEL |  |  |  |  |  |  |  |  |  |  |  |  |  |  |  |  |  |
| Borland  et al. ^34^ | 2020 | RCT | 97 | 74 | AF | NS | NS | ✓ | P | UC | UC | P | N | N | N | N | P | N | N |
| No acronym | SWEDEN | PARALLEL |  |  |  |  |  |  |  |  |  |  |  |  |  |  |  |  |  |
| Brinkmann  et al ^35^ | 2019 | RCT | 30 | 60 | DIABETES | NS | NS | ✓ | P | P | P | P | N | N | N | N | N | N | N |
| No acronym | GERMANY | PARALLEL |  |  |  |  |  |  |  |  |  |  |  |  |  |  |  |  |  |
| Callisaya  et al. ^36^ | 2017 | RCT | 50 | 66.2 | DIABETES | NS | NS | ✓ | P | P | P | P | N | N | Y | P | P | N | N |
| CDOT-X | AUSTRALIA | PARALLEL |  |  |  |  |  |  |  |  |  |  |  |  |  |  |  |  |  |
| Canning  et al. ^37^ | 2015 | RCT | 231 | 70.6 | PD + FALL HISTORY OR RISK OF FALL | NS | NS | ✓ | P | P | P | P | N | N | Y | P | P | N | N |
| PD-WEBB | AUSTRALIA | PARALLEL |  |  |  |  |  |  |  |  |  |  |  |  |  |  |  |  |  |
| Christensen et al. ^38^ | 2019 | QUASI-EX | 50 | 64.8 | CANCER | NS | NS | ✓ | P | P | P | P | N | N | Y | P | P | N | N |
| PRESET | DENMARK | FEASIBILITY STUDY |  |  |  |  |  |  |  |  |  |  |  |  |  |  |  |  |  |
| Christle  et al.^39^ | 2018 | RCT | 70 | 69.9 | REQUIRING CARDIAC REHAB | NS | NS | ✓ | P | P | P | P | N | N | N | N | N | N | N |
| DOPPELHERZ | GERMANY | PARALLEL |  |  |  |  |  |  |  |  |  |  |  |  |  |  |  |  |  |
| Englund  et al. ^40^ | 2018 | RCT | 149 | 77.4 | MOBILITY LIMITED OLDER ADULTS | 2.8 | NS | NA | P | P | P | P | N | N | N | P | P | N | N |
| VIVE2 | SWEDEN & USA | PARALLEL |  |  |  |  |  |  |  |  |  |  |  |  |  |  |  |  |  |
| Fabri  et al. ^41^ | 2019 | RCT | 28 | 63.5 | HEART FAILURE | NS | NS | ✓ | P | UC | UC | P | N | N | N | N | N | N | N |
| No acronym | BRAZIL | PARALLEL |  |  |  |  |  |  |  |  |  |  |  |  |  |  |  |  |  |
| Ferrer-García et al. ^42^ | 2011 | RCT | 84 | 66.6 | DIABETES | NS | NS | ✓ | P | UC | UC | P | N | N | N | N | P | N | N |
| No acronym | SPAIN | PARALLEL |  |  |  |  |  |  |  |  |  |  |  |  |  |  |  |  |  |
| Karjalainen  et al.^43^ | 2012 | RCT | 83 | 62 | CAD | NS | NS | ✓ | P | P | P | P | N | N | N | N | N | N | N |
| ARTEMIS | FINLAND | PARALLEL |  |  |  |  |  |  |  |  |  |  |  |  |  |  |  |  |  |
| Kolbe-Alexander  et al. ^44^ | 2006 | QUASI-EX | 81 | 67.5 | OLDER INACTIVE FEMALES | NS | NS | ✓ | P | P | P | P | N | N | N | P | P | N | N |
| CHIPs | SOUTH AFRICA | PRE-TEST-POST-TEST |  |  |  |  |  |  |  |  |  |  |  |  |  |  |  |  |  |
| Stubbs  et al. ^45^ | 2019 | RCT | 33 | 62.4 | DIABETES | NS | NS | ✓ | P | P | P | P | N | N | N | P | P | N | N |
| No acronym | USA | PARALLEL |  |  |  |  |  |  |  |  |  |  |  |  |  |  |  |  |  |
| Myers  et al. ^46^ | 2013 | RCT | 140 | 71.5 | AAA | NS | NS | ✓ | P | UC | UC | P | N | N | N | N | N | N | N |
| AAA STOP | USA | PARALLEL |  |  |  |  |  |  |  |  |  |  |  |  |  |  |  |  |  |
| Paulo  et al. ^47^ | 2019 | RCT | 36 | 64.9 | BREAST CANCER | NS | NS | ✓ | P | UC | UC | P | N | N | N | P | N | N | N |
| No acronym | BRAZIL | PARALLEL |  |  |  |  |  |  |  |  |  |  |  |  |  |  |  |  |  |
| Pressler  et al. ^48^ | 2016 | RCT | 30 | 81 | AORTIC VALVE DISEASE | NS | NS | ✓ | P | P | P | P | N | N | Y | P | P | N | N |
| SPORT: TAVI | GERMANY | PARALLEL |  |  |  |  |  |  |  |  |  |  |  |  |  |  |  |  |  |
| Ramponi  et al. ^49^ | 2013 | QUASI-EX | 27 | 69 | COPD | NS | NS | ✓ | P | P | P | P | N | N | Y | P | P | N | N |
| No acronym | Italy | PRE-TEST-POST-TEST |  |  |  |  |  |  |  |  |  |  |  |  |  |  |  |  |  |
| Rengo  et al. ^50^ | 2018 | QUASI-EX | 119 | 68 | HEART FAILURE | NS | NS | ✓ | P | UC | UC | P | N | N | N | P | P | N | N |
| No acronym | Canada | PRE-TEST-POST-TEST |  |  |  |  |  |  |  |  |  |  |  |  |  |  |  |  |  |
| Stanghelle  et al. ^51^ | 2020 | RCT | 149 | 74.2 | WOMEN WITH OSTEO-PEROSIS | NS | NS | ✓ | P | P | P | P | N | N | N | P | P | N | N |
| No acronym | NORWAY | PARALLEL |  |  |  |  |  |  |  |  |  |  |  |  |  |  |  |  |  |
| Thomas  et al. ^52^ | 2017 | RCT | 121 | 61.2 | INACTIVE FEMALES WITH CANCER | NS | NS | ✓ | P | P | P | P | N | N | N | P | P | N | N |
| HOPE | USA | PARALLEL |  |  |  |  |  |  |  |  |  |  |  |  |  |  |  |  |  |
| Torres-Sánchez  et al. ^53^ | 2017 | RCT | 90 | 71.7 | COPD | NS | 5.04 CCI | NA | P | UC | UC | P | N | N | N | P | P | N | N |
| No acronym | SPAIN | PARALLEL |  |  |  |  |  |  |  |  |  |  |  |  |  |  |  |  |  |
| Zaidi  et al. ^54^ | 2019 | RCT | 137 | 63.1 | DIABETES & CAD | NS | NS | ✓ | P | UC | P | P | N | N | N | P | P | N | N |
| EXCADI | NORWAY | PARALLEL |  |  |  |  |  |  |  |  |  |  |  |  |  |  |  |  |  |
| Barcellos  et al. ^55^ | 2018 | RCT | 150 | 65 | ↑BP & CKD | NS | NS | ✓ | P | UC | UC | P | N | N | N | P | P | N | N |
| No acronym | BRAZIL | PARALLEL |  |  |  |  |  |  |  |  |  |  |  |  |  |  |  |  |  |
| Berent  et al. ^56^ | 2011 | RCT | 295 | 62.7 | PRIOR CORONARY EVENT | NS | NS | ✓ | P | UC | UC | P | N | N | N | P | N | N | N |
| No acronym | AUSTRIA | PARALLEL |  |  |  |  |  |  |  |  |  |  |  |  |  |  |  |  |  |
| Edelmann  et al. ^57^ | 2011 | RCT | 64 | 65 | HEART FAILURE | NS | NS | ✓ | P | P | P | P | N | N | N | P | N | N | N |
| Ex-DHF-P | GERMANY | PARALLEL |  |  |  |  |  |  |  |  |  |  |  |  |  |  |  |  |  |
| Gary  et al. ^58^ | 2011 | RCT | 24 | 60 | HEART FAILURE | NS | 2.5 CCI | NA | P | UC | UC | P | N | N | N | P | P | N | N |
| No acronym | USA | PARALLEL |  |  |  |  |  |  |  |  |  |  |  |  |  |  |  |  |  |
| Casey  et al.^59^ | 2012 | RCT | 350 | 68.8 | COPD | NS | NS | ✓ | Y | Y | Y | Y | Y | P | N | Y | Y | Y | Y |
| PRINCE | IRELAND | CLUSTER |  |  |  |  |  |  |  |  |  |  |  |  |  |  |  |  |  |
| Nelson  et al. ^60^ | 2004 | RCT | 72 | 77.7 | OLDER ADULTS | 3.5 | NS | NA | P | P | P | P | N | N | N | P | P | N | N |
| No acronym | AUSTRALIA | PARALLEL |  |  |  |  |  |  |  |  |  |  |  |  |  |  |  |  |  |
| Helbostad  et al. ^61^ | 2004 | RCT | 77 | 81.1 | OLDER ADULTS WITH FRAILTY | 4.3 | NS | NA | P | UC | UC | P | N | N | N | N | N | N | N |
| No acronym | NORWAY | PARALLEL |  |  |  |  |  |  |  |  |  |  |  |  |  |  |  |  |  |

# Supplementary Table 4: Three component studies

| **AUTHORS &**  **ACRONYM** | **YEAR &**  **COUNTRY** | **DESIGN &**  **TYPE** | **SAMPLE SIZE**  **(n)** | **MEAN AGE**  **(YEARS)** | **SPECIFIC DISEASE TARGETED** | **COMORBIDITIES**  **(MEAN)** | **COMORBIDITY MEASURE (SCORE & NAME)** | **BASELINE PERCENTAGES OF CONCURRENT CHRONIC CONDITIONS SUGGEST MM** | **Plan the process** | **Involve Stakeholders** | **Bring together a team** | **Review published evidence** | **Draw on existing theories** | **Articulate programme theory** | **Undertake primary data collection** | **Understand context** | **Attend to future implementation** | **Design & Refine** | **End the Development Phase** |
| --- | --- | --- | --- | --- | --- | --- | --- | --- | --- | --- | --- | --- | --- | --- | --- | --- | --- | --- | --- |
| Fisher  et al.^62^ | 2018 | RCT | 172 | 60.3 | INACTIVITY | NS | NS | ✓ | P | UC | P | P | N | N | N | P | N | N | N |
| No acronym | CANADA | PARALLEL |  |  |  |  |  |  |  |  |  |  |  |  |  |  |  |  |  |
| Bernocchi  et al.^63^ | 2018 | RCT | 112 | 70.5 | COPD & HF | NS | NS | ✓ | P | UC | P | P | N | N | Y | P | N | N | N |
| No acronym | ITALY | PARALLEL |  |  |  |  |  |  |  |  |  |  |  |  |  |  |  |  |  |
| Hernandez  et al.^64^ | 2019 | RCT | 572 | 73 | ETHNIC MINORITY GROUPS | 2.6 | NA | NA | Y | P | Y | Y | Y | N | Y | Y | Y | UC | N |
| CAMINEMOS! | USA | PARALLEL |  |  |  |  |  |  |  |  |  |  |  |  |  |  |  |  |  |
| Roitto  et al.^65^ | 2018 | RCT | 210 | 78 | ALZHEIMER DISEASE | NS | 2.5  CCI | NA | P | P | Y | Y | N | N | N | P | N | N | N |
| FINALEX | FINLAND | PARALLEL |  |  |  |  |  |  |  |  |  |  |  |  |  |  |  |  |  |
| Selzler  et al.^66^ | 2016 | QUASI-EXP | 64 | 68.5 | COPD | NS | NS | ✓ | P | P | UC | P | Y | N | N | N | N | N | N |
| No acronym | CANADA | PRE-TEST POST-TEST |  |  |  |  |  |  |  |  |  |  |  |  |  |  |  |  |  |
| Shubert  et al.^67^ | 2020 | QUASI-EXP | 42 | 75 | FALLS RISK | 2.3 | NA | NA | P | UC | UC | Y | N | N | N | P | Y | N | N |
| STAND TALL | USA | PRE-TEST POST-TEST |  |  |  |  |  |  |  |  |  |  |  |  |  |  |  |  |  |
| Toots  et al.^68^ | 2019 | RCT | 186 | 85.1 | DEMENTIA | NS | NS | ✓ | P | P | P | P | N | N | Y | P | N | N | N |
| UMDEX | SWEDEN | CLUSTER |  |  |  |  |  |  |  |  |  |  |  |  |  |  |  |  |  |
| Van Dam van Isselt et al.^69^ | 2019 | QUASI-EXP | 159 | 70.8 | COPD | NS | NS | ✓ | Y | P | Y | Y | P | N | Y | P | P | N | Y |
| GR-COPD | NETHER-LANDS | PRE-TEST POST-TEST |  |  |  |  |  |  |  |  |  |  |  |  |  |  |  |  |  |

| Witham  et al.^70^ | 2008 | QUASI-EXP | 17 | 81.6 | HF | 5.1 | NA | NA | P | UC | UC | P | N | N | N | P | P | N | N |
| --- | --- | --- | --- | --- | --- | --- | --- | --- | --- | --- | --- | --- | --- | --- | --- | --- | --- | --- | --- |
| No acronym | UK | PRE-TEST POST-TEST |  |  |  |  |  |  |  |  |  |  |  |  |  |  |  |  |  |
| Angst  et al.^71^ | 2013 | QUASI-EXP | 252 | 65 | OSTEO-ARTHRITIS | NS | NS | ✓ | P | UC | UC | P | N | N | Y | N | N | N | N |
| No acronym | SWITZER-LAND | PRE-TEST POST-TEST |  |  |  |  |  |  |  |  |  |  |  |  |  |  |  |  |  |
| Gretebeck  et al.^72^ | 2019 | RCT | 111 | 70.8 | DIABETES | NS | NS | ✓ | P | UC | UC | P | N | N | N | P | P | N | N |
| No acronym | USA | PARALLEL |  |  |  |  |  |  |  |  |  |  |  |  |  |  |  |  |  |
| Hessert  et al.^73^ | 2005 | QUASI-EXP | 17 | 83.5 | CARE HOME RESIDENT | 3.7 | NA | NA | P | UC | UC | P | N | N | N | N | N | N | N |
| No acronym | USA | PRE-TEST POST-TEST |  |  |  |  |  |  |  |  |  |  |  |  |  |  |  |  |  |
| Lauze  et al.^74^ | 2017 | RCT | 32 | 81.1 | CARE HOME RESIDENT | 3.7 | NA | NA | Y | Y | P | P | N | N | Y | P | Y | N | N |
| No acronym | CANADA | PARALLEL |  |  |  |  |  |  |  |  |  |  |  |  |  |  |  |  |  |
| Marengoni  et al.^75^ | 2018 | RCT | 1260 | 69.1 | DEMENTIA RISK | 2 | NA | NA | Y | P | Y | P | N | Y | N | P | P | N | N |
| FINGER RCT | FINLAND | PARALLEL |  |  |  |  |  |  |  |  |  |  |  |  |  |  |  |  |  |
| Marigold  et al.^76^ | 2005 | RCT | 61 | 67.7 | STROKE | NS | NS | ✓ | P | UC | UC | P | N | N | N | N | N | N | N |
| No acronym | CANADA | PARALLEL |  |  |  |  |  |  |  |  |  |  |  |  |  |  |  |  |  |
| Underwood et al.^77^ | 2013 | RCT | 891 | 86.4 | CARE HOME RESIDENT | NS | NS | ✓ | P | P | P | Y | Y | N | N | Y | Y | UC | N |
| OPERA | UJ | CLUSTER |  |  |  |  |  |  |  |  |  |  |  |  |  |  |  |  |  |
| Wuthiwaropas et al.^78^ | 2013 | QUASI-EXP | 25 | 66 | CORONARY ARTERY DISEASE | NS | NS | ✓ | P | P | UC | P | N | N | N | N | N | N | N |
| No acronym | USA | PRE-TEST POST-TEST |  |  |  |  |  |  |  |  |  |  |  |  |  |  |  |  |  |
| Lin  et al.^79^ | 2007 | RCT | 150 | 76.8 | FALLERS | NS | NS | ✓ | P | UC | UC | P | N | N | N | P | N | N | N |
| No acronym | TAIWAN | PARALLEL |  |  |  |  |  |  |  |  |  |  |  |  |  |  |  |  |  |
| Robinson  et al.^80^ | 2002 | QUASI-EXP | 22 | 73.7 | OLDER ADULTS | NS | NS | ✓ | P | UC | UC | P | N | N | N | P | N | P | N |
| No acronym | USA | PRE-TEST POST-TEST |  |  |  |  |  |  |  |  |  |  |  |  |  |  |  |  |  |
| Suzuki  et al.^81^ | 2012 | RCT | 50 | 76 | COGNITIVE PROBLEMS | NS | NS | ✓ | P | P | UC | P | N | N | N | P | N | N | N |
| No acronym | JAPAN | PARALLEL |  |  |  |  |  |  |  |  |  |  |  |  |  |  |  |  |  |
| Brach  et al.^82^ | 2017 | RCT | 298 | 80 | CARE HOME RSIDENT | NS | 2.8 DCI | NA | Y | Y | Y | Y | N | N | Y | Y | Y | Y | Y |
| On The Move | USA | CLUSTER |  |  |  |  |  |  |  |  |  |  |  |  |  |  |  |  |  |
| Stevens-Lapsley  et al.^83^ | 2016 | RCT | 22 | 84.8 | HOME BOUND MULTI MORBID | 4.6 | NA | NA | P | P | P | P | N | N | N | P | P | N | N |
| No acronym | USA | PARALLEL |  |  |  |  |  |  |  |  |  |  |  |  |  |  |  |  |  |
| Greening  et al.^84^ | 2014 | RCT | 389 | 71.1 | COPD | 2 | NA | NA | P | P | P | P | N | N | N | N | P | N | N |
| REACH-trial | UK | PARALLEL |  |  |  |  |  |  |  |  |  |  |  |  |  |  |  |  |  |
| Pang  et al.^85^ | 2005 | RCT | 63 | 65.2 | STROKE | NS | NS | ✓ | P | P | UC | P | N | N | N | N | P | N | N |
| FAME | CANADA | PARALLEL |  |  |  |  |  |  |  |  |  |  |  |  |  |  |  |  |  |
| Brovold  et al. ^86^ | 2013 | RCT | 115 | 78 | POST HOSPITAL | 2.5 | NA | NA | P | P | P | P | N | N | N | P | P | N | N |
| No acronym | NORWAY | PARALLEL |  |  |  |  |  |  |  |  |  |  |  |  |  |  |  |  |  |
| de Rooij  et al.^87^ | 2017 | RCT | 126 | 63.5 | ONE CHRONIC DISEASE & OSTEO ARTHRITIS | NS | NS | ✓ | Y | Y | Y | Y | N | Y | Y | Y | Y | Y | Y |
| No acronym | NETHER-LANDS | PARALLEL |  |  |  |  |  |  |  |  |  |  |  |  |  |  |  |  |  |
| Andersson  et al.^88^ | 2020 | RCT | 74 | 69.6 | ARTHRITIS | NS | NS | ✓ | P | P | P | P | N | N | N | P | P | N | N |
| PEP-walk | SWEDEN | PARALLEL |  |  |  |  |  |  |  |  |  |  |  |  |  |  |  |  |  |
| Zak  et al.^89^ | 2006 | RCT | 50 | 87.1 | FREQUENT FALLS | 4.4 | NA | NA | P | UC | UC | UC | N | N | N | N | N | N | N |
| No acronym | POLAND | PARALLEL |  |  |  |  |  |  |  |  |  |  |  |  |  |  |  |  |  |
| Gaunaurd  et al.^90^ | 2014 | RCT | 22 | 68.6 | PULMON-ARY FIBROSIS | NS | NS | ✓ | P | UC | UC | P | N | N | N | P | N | N | N |
| No acronym | USA | PARALLEL |  |  |  |  |  |  |  |  |  |  |  |  |  |  |  |  |  |
| Gill  et al.^91^ | 2004 | RCT | 188 | 83.1 | FRAILTY | 2 | NA | NA | Y | Y | Y | Y | Y | N | Y | P | P | Y | Y |
| PREHAB | USA | PARALLEL |  |  |  |  |  |  |  |  |  |  |  |  |  |  |  |  |  |
| Kovacs et al.^92^ | 2013 | RCT | 76 | 68.4 | OLDER FEMALES | NS | NS | ✓ | P | UC | UC | P | N | N | N | N | N | N | N |
| No acronym | HUNGARY | PARALLEL |  |  |  |  |  |  |  |  |  |  |  |  |  |  |  |  |  |
| Santos  et al.^93^ | 2015 | RCT | 76 | 68.4 | COPD | NS | NS | ✓ | P | P | P | P | N | N | N | N | N | N | N |
| No acronym | PORTUGAL | PARALLEL |  |  |  |  |  |  |  |  |  |  |  |  |  |  |  |  |  |
| Morano  et al.^94^ | 2013 | RCT | 24 | 66.8 | CANCER | NS | NS | ✓ | P | UC | UC | P | N | N | N | N | N | N | N |
| No acronym | BRAZIL | PARALLEL |  |  |  |  |  |  |  |  |  |  |  |  |  |  |  |  |  |
| Kim  et al.^95^ | 2016 | RCT | 105 | 81.1 | SARCO-PAENIC OBESITY | NS | NS | ✓ | P | UC | UC | P | N | N | N | P | N | N | N |
| No acronym | JAPAN | PARALLEL |  |  |  |  |  |  |  |  |  |  |  |  |  |  |  |  |  |
| VanSwearing et al.^96^ | 2011 | RCT | 50 | 77.2 | MOBILITY PROBLEMS | 2.6 | NA | NA | P | P | P | P | N | N | N | P | P | N | N |
| No acronym | USA | PARALLEL |  |  |  |  |  |  |  |  |  |  |  |  |  |  |  |  |  |
| Villareal  et al.^97^ | 2006 | RCT | 27 | 70 | OBESE INACTIVE ADULTS | 2 | NA | NA | P | P | P | P | Y | N | N | P | P | N | N |
| No acronym | USA | PARALLEL |  |  |  |  |  |  |  |  |  |  |  |  |  |  |  |  |  |
| Yang  et al.^98^ | AUS-TRAILIA | RCT | 165 | 80.5 | ADULTS WITH BALANCE PROBLEMS | 4 | NA | NA | P | P | P | P | N | N | N | P | P | N | N |
| No acronym | 2012 | PARALEL |  |  |  |  |  |  |  |  |  |  |  |  |  |  |  |  |  |
| Wisniowska et al.^99^ | POLAND | RCT | 204 | 74.2 | INACTIVE ADULTS | NS | NS | ✓ | P | UC | UC | P | P | N | N | P | N | N | N |
| No acronym | 2020 | PARALLEL |  |  |  |  |  |  |  |  |  |  |  |  |  |  |  |  |  |
| Echeverria  et al.^100^ | SPAIN | RCT | 55 | 82.5 | OLDER ADULT IN HOSPITAL | NS | 5.7 CCI | NAP | P | UC | UC | P | N | N | N | P | P | N | N |
| No acronym | 2020 | PARALLEL |  |  |  |  |  |  |  |  |  |  |  |  |  |  |  |  |  |
| Hewitt  et al.^101^ | 2018 | RCT | 221 | 86 | CARE HOME RESIDENT | NS | NS | ✓ | P | P | P | P | N | N | N | P | P | N | N |
| Sunbeam | AUS-TRALIA | CLUSTER |  |  |  |  |  |  |  |  |  |  |  |  |  |  |  |  |  |
| Rubenstein  et al.^102^ | 2000 | RCT | 59 | 75.4 | OLDER MEN WITH FALLS RISK | NS | NS | ✓ | P | UC | UC | UC | N | N | N | N | N | N | N |
| No acronym | USA | PARALLEL |  |  |  |  |  |  |  |  |  |  |  |  |  |  |  |  |  |

# Supplementary Table 5: Four component studies

| **AUTHORS &**  **ACRONYM** | **YEAR &**  **COUNTRY** | **DESIGN &**  **TYPE** | **SAMPLE SIZE**  **(n)** | **MEAN AGE**  **(YEARS)** | **SPECIFIC DISEASE TARGETED** | **COMORBIDITIES**  **(MEAN)** | **COMORBIDITY MEASURE (SCORE & NAME)** | **BASELINE PERCENTAGES OF CONCURRENT CHRONIC CONDITIONS SUGGEST MM** | **Plan the process** | **Involve Stakeholders** | **Bring together a team** | **Review published evidence** | **Draw on existing theories** | **Articulate programme theory** | **Undertake primary data collection** | **Understand context** | **Attend to future implementation** | **Design & Refine** | **End the Development Phase** |
| --- | --- | --- | --- | --- | --- | --- | --- | --- | --- | --- | --- | --- | --- | --- | --- | --- | --- | --- | --- |
| Serra-Prat  et al.^103^ | 2017 | RCT | 172 | 78.3 | FRAILTY | 3.6 | NA | NA | P | P | UC | P | N | N | N | N | N | N | N |
| No acronym | SPAIN | PARALLEL |  |  |  |  |  |  |  |  |  |  |  |  |  |  |  |  |  |
| Matcher  et al.^104^ | 2017 | RCT | 354 | 77.7 | FALLS | NS | NS | ✓ | P | P | P | P | N | N | N | P | P | N | N |
| SAFE | SINGA-PORE | PARALLEL |  |  |  |  |  |  |  |  |  |  |  |  |  |  |  |  |  |
| Reeves  et al.^105^ | 2017 | RCT | 27 | 72.3 | HEART FAILURE | 4.9 | NA | NA | P | P | P | P | N | N | Y | P | P | N | N |
| REHAB-HF (pilot) | USA | PARALEL |  |  |  |  |  |  |  |  |  |  |  |  |  |  |  |  |  |
| Baptista  et al.^106^ | 2018 | QUASI EXP | 96 | 67.4 | HYPER-TENSION | 2.3 | NA | NA | P | N | P | P | N | N | N | N | N | N | N |
| No acronym | PORTUGAL | PRE-TEST POST-TEST |  |  |  |  |  |  |  |  |  |  |  |  |  |  |  |  |  |
| Baptista  et al^107^. | 2017 | QUASI EXP | 284 | 70.2 | DIABETES | 2 | NA | NA | P | UC | UC | P | N | N | N | N | N | N | N |
| No acronym | PORTUGAL | PRE-TEST POST-TEST |  |  |  |  |  |  |  |  |  |  |  |  |  |  |  |  |  |
| Tomioka  et al.^108^ | 2019 | QUASI EXP | 1202 | 85.5% >65 | OLDER ADULTS | NS | NS | ✓ | P | P | UC | P | N | N | N | P | N | N | N |
| Enhance Fitness | HAWAII | PRE-TEST POST-TEST |  |  |  |  |  |  |  |  |  |  |  |  |  |  |  |  |  |
| Vahlberg  et al.^109^ | 2016 | RCT | 43 | 73.2 | STROKE | NS | NS | ✓ | P | P | UC | P | N | N | N | P | PN | N | N |
| No acronym | SWEDEN | PARALLEL |  |  |  |  |  |  |  |  |  |  |  |  |  |  |  |  |  |
| Gunther  et al.^110^ | 2003 | QUASI EXP | 36 | 73 | ARTHRITIS | NS | NS | ✓ | P | UC | UC | P | N | N | N | N | N | N | N |
| No acronym | USA | PRE-TEST POST-TEST |  |  |  |  |  |  |  |  |  |  |  |  |  |  |  |  |  |
| Hsieh  et al.^111^ | 2019 | RCT | 236 | 71.5 | FRAILTY | NS | NS | ✓ | P | P | P | P | N | N | N | P | N | N | N |
| No acronym | TAIWAN | PARALLEL |  |  |  |  |  |  |  |  |  |  |  |  |  |  |  |  |  |
| Loyola et al.^112^ | 2018 | QUASI EXP | 164 | 71.7 | OLDER ADULTS | NS | 3.1 CCI | NA | P | P | P | P | N | N | N | P | N | N | N |
| No acronym | CHILE | PRE-TEST POST-TEST |  |  |  |  |  |  |  |  |  |  |  |  |  |  |  |  |  |
| Schlenk  et al.^113^ | 2020 | RCT | 182 | 64.7 | OSTEO-ARTHRITIS & HYPER-TENSION | 6.6 | NA | NA | P | P | P | P | Y | N | Y | Y | Y | P | N |
| STAR | USA | PARALLEL |  |  |  |  |  |  |  |  |  |  |  |  |  |  |  |  |  |
| Smith  et al.^114^ | 2006 | QUASI EXP | 113 | 76 | ≥3 CHRONIC DISEASES | NS | NS | ✓ | P | P | P | P | N | N | N | P | N | N | N |
| No acronym | USA | PRE-TEST POST-TEST |  |  |  |  |  |  |  |  |  |  |  |  |  |  |  |  |  |
| Hinrichs  et al.^115^ | 2015 | RCT | 209 | 79.8 | INACTIVE ADULTS WITH MULTI-MORBIDITY | PNPSP | PNNSN | Y✓P | P | P | P | P | N | N | Y | P | P | N | N |
| HOMEfit | SWITZER-LAND | PARALLEL |  |  |  |  |  |  |  |  |  |  |  |  |  |  |  |  |  |
| Cesari  et al.^116^ | 2015 | RCT | 424 | 76.6 | INACTIVE OLDER ADULTS | NS | NS | ✓ | P | P | Y | P | Y | N | Y | Y | Y | UC | N |
| LIFE-P | USA | PARALLEL |  |  |  |  |  |  |  |  |  |  |  |  |  |  |  |  |  |
| Courtney  et al^117^ | 2009 | RCT | 128 | 78.8 | AT RISK OF REPEAT HOSP. | 5 | NA | NA | P | P | P | P | N | N | N | P | N | N | N |
| No acronym | AUSTRALIA | PARALLEL |  |  |  |  |  |  |  |  |  |  |  |  |  |  |  |  |  |
| Tse  et al.^118^ | 2011 | QUASI EXP | 75 | 84.9 | NURSING HOME RESIDENT | NS | NS | ✓ | P | UC | UC | P | N | N | N | P | N | N | N |
| No acronym | CHINA | PRE-TEST POST-TEST |  |  |  |  |  |  |  |  |  |  |  |  |  |  |  |  |  |
| Aquino  et al.^119^ | 2016 | RCT | 28 | 67.2 | COPD | NS | NS | ✓ | P | UC | UC | P | N | N | N | N | N | N | N |
| No acronym | ITALY | PARALLEL |  |  |  |  |  |  |  |  |  |  |  |  |  |  |  |  |  |
| Boongird  et al. ^120^ | 2017 | RCT | 439 | 74 | BALANCE PORBLEMS | NS | NS | ✓ | P | Y | Y | P | N | N | Y | P | Y | Y | N |
| No acronym | THAILAND | PARALLEL |  |  |  |  |  |  |  |  |  |  |  |  |  |  |  |  |  |
| Bouchonville et al.^121^ | 2014 | RCT | 80 | 69.7 | OBESITY & FRAILTY | 2 | NA | NA | P | P | P | P | N | N | Y | P | P | N | N |
| No acronym | USA | PARALLEL |  |  |  |  |  |  |  |  |  |  |  |  |  |  |  |  |  |
| Campo  et al.^122^ | 2020 | RCT | 235 | 76.4 | ACUTE CORONARY SYN-DROME | NS | NS | ✓ | P | P | P | P | N | N | N | N | P | N | N |
| HULK | ITALY | PARALLEL |  |  |  |  |  |  |  |  |  |  |  |  |  |  |  |  |  |
| Edgren  et al.^123^ | 2015 | RCT | 81 | 79.4 | HIP FRACTURE | 3 | NA | NA | P | P | P | P | N | N | N | P | P | N | N |
| ProMo | FINLAND | PARALLEL |  |  |  |  |  |  |  |  |  |  |  |  |  |  |  |  |  |
| Kwok  et al. ^124^ | 2016 | RCT | 80 | 70.1 | FRAILTY | NS | NS | ✓ | P | P | P | P | N | N | N | P | P | N | N |
| EFFECT | SINGA-PORE | PARALLEL |  |  |  |  |  |  |  |  |  |  |  |  |  |  |  |  |  |
| Newman  et al.^125^ | 2014 | RCT | 1635 | 78.8 | INACTIVE WITH MOBILITY PROBLEM | NS | NS | ✓ | Y | Y | Y | P | Y | N | Y | P | P | UC | N |
| LIFE | USA | PARALLEL |  |  |  |  |  |  |  |  |  |  |  |  |  |  |  |  |  |
| Perula  et al.^126^ | 2012 | RCT | 404 | 76.4 | OLDER ADULTS | 3 | NA | NA | P | Y | Y | P | N | N | N | P | N | N | N |
| EPICA | SPAIN | CLUSTER |  |  |  |  |  |  |  |  |  |  |  |  |  |  |  |  |  |
| Piva  et al.^127^ | 2019 | RCT | 240 | 69.6 | KNEE REPLACE & MOBILITY PROBLEMS | 4.4 | NA | NA | Y | Y | Y | Y | N | N | N | Y | Y | N | N |
| No acronym | USA | PARALLEL |  |  |  |  |  |  |  |  |  |  |  |  |  |  |  |  |  |
| Vainshelboim et al.^128^ | 2016 | RCT | 32 | 67.3 | PULMON-ARY FIBROSIS | NS | NS | ✓ | P | UC | UC | P | N | N | N | P | P | N | N |
| No acronym | ISREAL | PARALLEL |  |  |  |  |  |  |  |  |  |  |  |  |  |  |  |  |  |
| Venkataraman et al.^129^ | 2019 | RCT | 143 | 62.1 | DIABETES | NS | NS | ✓ | P | P | P | P | N | N | Y | P | P | N | N |
| PDN-QoL | SINGA-PORE | PARALLEL |  |  |  |  |  |  |  |  |  |  |  |  |  |  |  |  |  |
| Villareal  et al.^130^ | 2017 | RCT | 160 | 70 | INACTIVE ADULTS WITH OBESITY & FRAILTY | 3 | NA | NA | Y | P | P | P | N | N | Y | P | P | N | N |
| LITOE | USA | PARALLEL |  |  |  |  |  |  |  |  |  |  |  |  |  |  |  |  |  |
| Batra  et al.^131^ | 2019 | QUASI EXP | 1295 | ALL >60 | OLDER ADULTS | NS | NS | ✓ |  |  |  |  |  |  |  |  |  |  |  |
| Enhance Fitness | USA | PRE-TEST POST-TEST |  |  |  |  |  |  |  |  |  |  |  |  |  |  |  |  |  |
| Barnett  et al.^132^ | 2003 | RCT | 163 | 74.8 | FALLS RISK | NS | NS | ✓ | P | P | P | P | N | N | N | P | P | N | N |
| No acronym | AUSTRALIA | PARALLEL |  |  |  |  |  |  |  |  |  |  |  |  |  |  |  |  |  |
| Comans  et al.^133^ | 2010 | RCT | 107 | 78.9 | FALLS RISK | 3.9 | NA | NA | P | P | P | P | N | N | N | P | N | N | N |
| No acronym | AUSTRALIA | PARALLEL |  |  |  |  |  |  |  |  |  |  |  |  |  |  |  |  |  |
| Binder  et al.^134^ | 2002 | RCT | 119 | 83 | FRAILTY | NS | NS | ✓ | P | UC | UC | P | N | N | N | N | N | N | N |
| No acronym | USA | PARALLEL |  |  |  |  |  |  |  |  |  |  |  |  |  |  |  |  |  |

# Supplementary Table 6: Five component studies

| **AUTHORS &**  **ACRONYM** | **YEAR &**  **COUNTRY** | **DESIGN &**  **TYPE** | **SAMPLE SIZE**  **(n)** | **MEAN AGE**  **(YEARS)** | **SPECIFIC DISEASE TARGETED** | **COMORBIDITIES**  **(MEAN)** | **COMORBIDITY MEASURE (SCORE & NAME)** | **BASELINE PERCENTAGES OF CONCURRENT CHRONIC CONDITIONS SUGGEST MM** | **Plan the process** | **Involve Stakeholders** | **Bring together a team** | **Review published evidence** | **Draw on existing theories** | **Articulate programme theory** | **Undertake primary data collection** | **Understand context** | **Attend to future implementation** | **Design & Refine** | **End the Development Phase** |
| --- | --- | --- | --- | --- | --- | --- | --- | --- | --- | --- | --- | --- | --- | --- | --- | --- | --- | --- | --- |
| Yokocji  et al.^135^ | 2012 | QUASI  EXP | 61 | 68.3 | ARTHRITIS + OBESITY OR MS OR DIABETES | NS | NS | ✓ | P | P | P | P | N | N | N | N | N | N | N |
| No acronym | JAPAN | PRE-TEST POST-TEST |  |  |  |  |  |  |  |  |  |  |  |  |  |  |  |  |  |
| Morey  et al.^136^ | 1989 | QUASI  EXP | 69 | ~70 | VETERANS | NS | NS | ✓ | P | P | UC | P | N | N | N | N | N | N | N |
| No acronym | USA | PRE-TEST POST-TEST |  |  |  |  |  |  |  |  |  |  |  |  |  |  |  |  |  |
| Tarazone  et al.^137^ | 2016 | RCT | 100 | 79.9 | INACTIVE FRAIL OLDER ADULTS | NS | NS | ✓ | P | P | P | P | N | N | N | P | P | N | N |
| EMTIFE | SPAIN | PARALLEL |  |  |  |  |  |  |  |  |  |  |  |  |  |  |  |  |  |
| Kalka  et al.^138^ | 2013 | RCT | 286 | 61.5 | ISCHAEMIC HEART DISEASE | NS | NS | ✓ | P | UC | UC | P | N | N | N | N | N | N | N |
| No acronym | POLAND | PARALLEL |  |  |  |  |  |  |  |  |  |  |  |  |  |  |  |  |  |

# Supplementary Figure 1: ROBINS-I

**
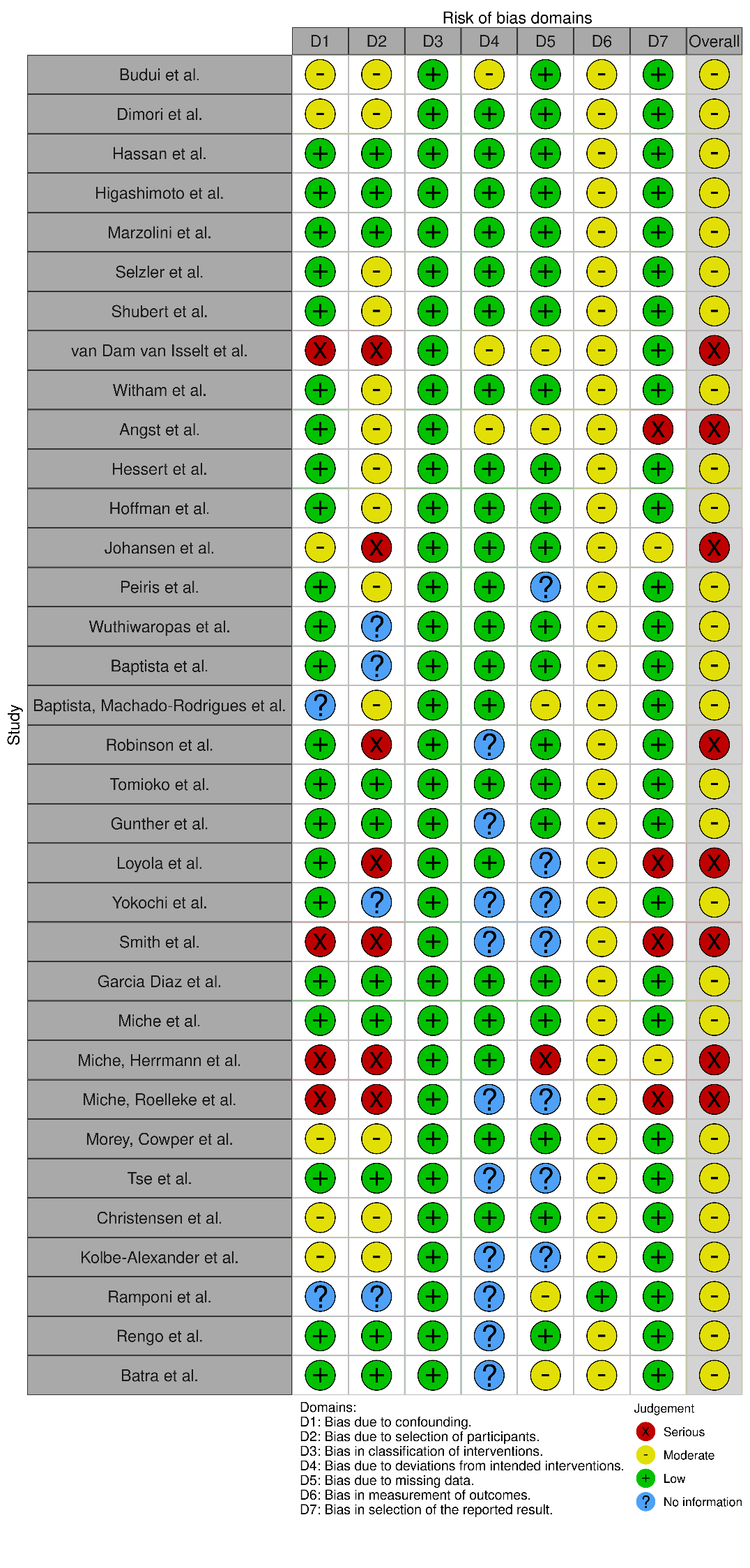
**

# Supplementary Figure 2: Cochrane Risk of Bias 2 Plot

# References

1. Bourne S, Devos R, North M, Chauhan A, Green B, Brown T, Cornelius V, Wilkinson T. Online versus face-to-face pulmonary rehabilitation for patients with chronic obstructive pulmonary disease: Randomised controlled trial. *BMJ Open* 2017;**7 (7) (no pagination)**. doi: <http://dx.doi.org/10.1136/bmjopen-2016-014580>

2. Budui S, Bigolin F, Giordano F, Leoni S, Berteotti M, Sartori E, Franceschini L, Taddei M, Salvetti S, Castiglioni F, Gilli F, Skafidas S, Schena F, Letizia Petroni M, Busetto L. Effects of an intensive inpatient rehabilitation program in elderly patients with obesity. *Obesity Facts* 2019;**12**:199-210. doi: <http://dx.doi.org/10.1159/000497461>

3. Dimori S, Leoni G, Fior L, Gasparotto F. Clinical nutrition and physical rehabilitation in a long-term care setting: preliminary observations in sarcopenic older patients. *Aging Clinical and Experimental Research* 2018;**30**:951-958. doi: <http://dx.doi.org/10.1007/s40520-017-0859-8>

4. Hassan M, Mourad S, Abdel Wahab NH, Daabis R, Younis G. Effect of comorbidities on response to pulmonary rehabilitation in patients with chronic obstructive pulmonary disease. *Egyptian Journal of Chest Diseases and Tuberculosis* 2016;**65**:63-69. doi: <http://dx.doi.org/10.1016/j.ejcdt.2015.11.006>

5. Higashimoto Y, Yamagata T, Maeda K, Honda N, Sano A, Nishiyama O, Sano H, Iwanaga T, Chiba Y, Fukuda K, Tohda Y. Influence of comorbidities on the efficacy of pulmonary rehabilitation in patients with chronic obstructive pulmonary disease. *Geriatrics and Gerontology International* 2016;**16**:934-941. doi: <http://dx.doi.org/10.1111/ggi.12575>

6. Marzolini S, Danells C, Oh PI, Jagroop D, Brooks D. Feasibility and Effects of Cardiac Rehabilitation for Individuals after Transient Ischemic Attack. *Journal of Stroke and Cerebrovascular Diseases* 2016;**25**:2453-2463. doi: <http://dx.doi.org/10.1016/j.jstrokecerebrovasdis.2016.06.018>

7. Allen JD, Vanbruggen MD, Johannsen NM, Robbins JL, Credeur DP, Pieper CF, Sloane R, Earnest CP, Church TS, Ravussin E, Kraus WE, Welsch MA. PRIME: A Novel Low-Mass, High-Repetition Approach to Improve Function in Older Adults. *Medicine and Science in Sports and Exercise* 2018;**50**:1005-1014. doi: <http://dx.doi.org/10.1249/MSS.0000000000001518>

8. Chen KM, Li CH, Huang HT, Cheng YY. Feasible modalities and long-term effects of elastic band exercises in nursing home older adults in wheelchairs: A cluster randomized controlled trial. *International Journal of Nursing Studies* 2016;**55**:4-14. doi: <http://dx.doi.org/10.1016/j.ijnurstu.2015.11.004>

9. Giné-Garriga M, Martin-Borràs C, Puig-Ribera A, Martín-Cantera C, Solà M, Cuesta-Vargas A. The Effect of a Physical Activity Program on the Total Number of Primary Care Visits in Inactive Patients: a 15-Month Randomized Controlled Trial. *PloS one* 2013;**8**:e66392. doi: 10.1371/journal.pone.0066392

10. Hellberg M, Hoglund P, Svensson P, Clyne N. Comparing effects of 4 months of two self-administered exercise training programs on physical performance in patients with chronic kidney disease: RENEXC - A randomized controlled trial. *PLoS ONE* 2018;**13 (12) (no pagination)**. doi: <http://dx.doi.org/10.1371/journal.pone.0207349>

11. Hoffman AJ, Brintnall RA, von Eye A, Jones LW, Alderink G, Patzelt LH, Brown JK. Home-based exercise: Promising rehabilitation for symptom relief, improved functional status and quality of life for post-surgical lung cancer patients. *Journal of Thoracic Disease* 2014;**6**:632-640. doi: <http://dx.doi.org/10.3978/j.issn.2072-1439.2014.06.08>

12. Johansen I, Lindbaek M, Stanghelle JK, Brekke M. Structured community-based inpatient rehabilitation of older patients is better than standard primary health care rehabilitation: an open comparative study. *Disability and rehabilitation* 2012;**34**:2039-2046. doi:

13. Peiris CL, Taylor NF, Hull S, Anderson A, Belski R, Fourlanos S, Shields N. A Group Lifestyle Intervention Program Is Associated with Reduced Emergency Department Presentations for People with Metabolic Syndrome: A Retrospective Case-Control Study. *Metabolic Syndrome and Related Disorders* 2018;**16**:110-116. doi: <http://dx.doi.org/10.1089/met.2017.0127>

14. Sajatovic M, Ridgel AL, Walter EM, Tatsuoka CM, Colon-Zimmermann K, Ramsey RK, Welter E, Gunzler SA, Whitney CM, Walter BL. A randomized trial of individual versus group-format exercise and self-management in individuals with Parkinson's disease and comorbid depression. *Patient Preference and Adherence* 2017;**11**:965-973. doi: <http://dx.doi.org/10.2147/PPA.S135551>

15. Zgibor JC, Ye L, Boudreau RM, Conroy MB, Vander Bilt J, Rodgers EA, Schlenk EA, Jacob ME, Brandenstein J, Albert SM, et al. Community-Based Healthy Aging Interventions for Older Adults with Arthritis and Multimorbidity. *Journal of community health* 2017;**42**:390‐399. doi: 10.1007/s10900-016-0268-5

16. Boxall A, Barclay L, Sayers A, Caplan GA. Managing chronic obstructive pulmonary disease in the community: a randomized controlled trial of home-based pulmonary rehabilitation for elderly housebound patients. *Journal of Cardiopulmonary Rehabilitation* 2005;**25**:378-385. doi:

17. Morey MC, Zhu CW. Improved fitness narrows the symptom-reporting gap between older men and women. *Journal of Women's Health (15409996)* 2003;**12**:381-390. doi: 10.1089/154099903765448899

18. Serra-Rexach JA, Bustamante-Ara N, Hierro Villarán M, González Gil P, Sanz Ibáñez MJ, Blanco Sanz N, Ortega Santamaría V, Gutiérrez Sanz N, Marín Prada AB, Gallardo C, et al. Short-term, light- to moderate-intensity exercise training improves leg muscle strength in the oldest old: a randomized controlled trial. *Journal of the american geriatrics society* 2011;**59**:594‐602. doi: 10.1111/j.1532-5415.2011.03356.x

19. Hetherington S, Henwood T, Swinton P, Keogh J, Gardiner P, Tuckett A, Rouse K. Engineering Improved Balance Confidence in Older Adults With Complex Health Care Needs: Learning From the Muscling Up Against Disability Study. *Archives of Physical Medicine & Rehabilitation* 2018;**99**:1525-1532. doi: 10.1016/j.apmr.2018.03.004

20. Caminiti G, Volterrani M, Marazzi G, Cerrito A, Massaro R, Sposato B, Arisi A, Rosano G. Hydrotherapy added to endurance training versus endurance training alone in elderly patients with chronic heart failure: a randomized pilot study. *International Journal of Cardiology* 2011;**148**:199-203. doi: <https://dx.doi.org/10.1016/j.ijcard.2009.09.565>

21. Chen MS, Lin TC, Jiang BC. Aerobic and resistance exercise training program intervention for enhancing gait function in elderly and chronically ill Taiwanese patients. *Public Health* 2015;**129**:1114-1124. doi: <https://dx.doi.org/10.1016/j.puhe.2015.04.018>

22. Erbs S, Hollriegel R, Linke A, Beck EB, Adams V, Gielen S, Mobius-Winkler S, Sandri M, Krankel N, Hambrecht R, Schuler G. Exercise training in patients with advanced chronic heart failure (NYHA IIIb) promotes restoration of peripheral vasomotor function, induction of endogenous regeneration, and improvement of left ventricular function. *Circulation: Heart Failure* 2010;**3**:486-494. doi: <https://dx.doi.org/10.1161/CIRCHEARTFAILURE.109.868992>

23. Garcia Diaz E, Alonso Ramirez J, Herrera Fernandez N, Peinado Gallego C, Perez Hernandez DG. Effect of strength exercise with elastic bands and aerobic exercise in the treatment of frailty of the elderly patient with type 2 diabetes mellitus. *Endocrinologia Diabetes y Nutricion* 2019;**66**:563-570. doi: <https://dx.doi.org/10.1016/j.endinu.2019.01.010>

24. Gielen S, Sandri M, Kozarez I, Kratzsch J, Teupser D, Thiery J, Erbs S, Mangner N, Lenk K, Hambrecht R, Schuler G, Adams V. Exercise training attenuates MuRF-1 expression in the skeletal muscle of patients with chronic heart failure independent of age: the randomized Leipzig Exercise Intervention in Chronic Heart Failure and Aging catabolism study. *Circulation* 2012;**125**:2716-2727. doi: <https://dx.doi.org/10.1161/CIRCULATIONAHA.111.047381>

25. Hegbom F, Sire S, Heldal M, Orning OM, Stavem K, Gjesdal K. Short-term exercise training in patients with chronic atrial fibrillation: effects on exercise capacity, AV conduction, and quality of life. *Journal of Cardiopulmonary Rehabilitation* 2006;**26**:24-29. doi:

26. Miche E, Herrmann G, Nowak M, Wirtz U, Tietz M, Hurst M, Zoller B, Radzewitz A. Effect of an exercise training program on endothelial dysfunction in diabetic and non-diabetic patients with severe chronic heart failure. *Clinical Research in Cardiology* 2006;**95 Suppl 1**:i117-124. doi:

27. Miche E, Herrmann G, Wirtz U, Laki H, Barth M, Radzewitz A. Effects of education, self-care instruction and physical exercise on patients with chronic heart failure. *Zeitschrift fur Kardiologie* 2003;**92**:985-993. doi:

28. Miche E, Roelleke E, Wirtz U, Zoller B, Tietz M, Huerst M, Radzewitz A. Combined endurance and muscle strength training in female and male patients with chronic heart failure. *Clinical Research in Cardiology* 2008;**97**:615-622. doi: <https://dx.doi.org/10.1007/s00392-008-0660-y>

29. Padberg FT, Jr., Johnston MV, Sisto SA. Structured exercise improves calf muscle pump function in chronic venous insufficiency: a randomized trial. *Journal of Vascular Surgery* 2004;**39**:79-87. doi:

30. Thompson RF, Crist DM, Marsh M, Rosenthal M. Effects of physical exercise for elderly patients with physical impairments. *Journal of the American Geriatrics Society* 1988;**36**:130-135. doi:

31. Yu DS, Lee DT, Woo J, Hui E. Non-pharmacological interventions in older people with heart failure: effects of exercise training and relaxation therapy. *Gerontology* 2007;**53**:74-81. doi:

32. Barker K, Holland AE, Lee AL, Haines T, Ritchie K, Boote C, Saliba J, Lowe S, Pazsa F, Thomas L, Turczyniak M, Skinner EH. Multimorbidity rehabilitation versus disease-specific rehabilitation in people with chronic diseases: A pilot randomized controlled trial. *Pilot and Feasibility Studies* 2018;**4**. doi: <http://dx.doi.org/10.1186/s40814-018-0369-2>

33. Silva Narlon CBS, Gregory Michael A, Gill Dawn P, Petrella Robert J. Multiple-modality exercise and mind-motor training to improve cardiovascular health and fitness in older adults at risk for cognitive impairment: a randomized controlled trial. *Archives of gerontology and geriatrics* 2017:149‐160. doi: 10.1016/j.archger.2016.10.009

34. Borland M, Bergfeldt L, Nordeman L, Bollano E, Andersson L, Rosenkvist A, Jakobsson M, Olsson K, Corin M, Landh L, et al. Exercise-based cardiac rehabilitation improves physical fitness in patients with permanent atrial fibrillation – A randomized controlled study. *Translational sports medicine* 2020. doi: 10.1002/tsm2.166

35. Brinkmann C, Weh-Gray O, Brixius K, Bloch W, Predel HG, Kreutz T. Effects of exercising before breakfast on the health of T2DM patients-A randomized controlled trial. *Scandinavian journal of medicine & science in sports* 2019;**29**:1930‐1936. doi: 10.1111/sms.13543

36. Callisaya ML, Daly RM, Sharman JE, Bruce D, Davis TME, Greenaway T, Nolan M, Beare R, Schultz MG, Phan T, et al. Feasibility of a multi-modal exercise program on cognition in older adults with Type 2 diabetes - a pilot randomised controlled trial. *BMC geriatrics* 2017;**17**:237. doi: 10.1186/s12877-017-0635-9

37. Canning CG, Sherrington C, Lord SR, Close JC, Heritier S, Heller GZ, Howard K, Allen NE, Latt MD, Murray SM, et al. Exercise for falls prevention in Parkinson disease: a randomized controlled trial. *Neurology* 2015;**84**:304‐312. doi: 10.1212/WNL.0000000000001155

38. Christensen JF, Simonsen C, Banck-Petersen A, Thorsen-Streit S, Herrstedt A, Djurhuus SS, Egeland C, Mortensen CE, Kofoed SC, Kristensen TS, et al. Safety and feasibility of preoperative exercise training during neoadjuvant treatment before surgery for adenocarcinoma of the gastro-oesophageal junction. *BJS open* 2019;**3**:74‐84. doi: 10.1002/bjs5.50110

39. Christle JW, Schlumberger A, Zelger O, Haller B, Beckers P, Myers J, Halle M, Pressler A. Effect of Individualized Combined Exercise Versus Group-Based Maintenance Exercise in Patients With Heart Disease and Reduced Exercise Capacity: THE DOPPELHERZ TRIAL. *Journal of cardiopulmonary rehabilitation and prevention* 2018;**38**:31‐37. doi: 10.1097/HCR.0000000000000306

40. Englund D, Kirn D, Koochek A, Travison T, Reid K, Von Berens A, Zhu H, Lilja M, Gustafsson T, Cederholm T, et al. Nutritional supplementation with physical activity improves muscle composition in mobility-limited older adults, the VIVE2 study: a randomized, double-blind, placebo-controlled trial. *FASEB journal* 2017;**31**. doi:

41. Fabri T, Catai AM, Ribeiro FHO, Junior JAA, Milan-Mattos J, Rossi DAA, Coneglian RC, Borra RC, Bazan SGZ, Hueb JC, et al. Impact of a supervised twelve-week combined physical training program in heart failure patients: a randomized trial. *Cardiology research and practice* 2019;**2019**. doi: 10.1155/2019/1718281

42. Ferrer-García JC, Sánchez López P, Pablos-Abella C, Albalat-Galera R, Elvira-Macagno L, Sánchez-Juan C, Pablos-Monzó A. Benefits of a home-based physical exercise program in elderly subjects with type 2 diabetes mellitus. *Endocrinologia y nutricion* 2011;**58**:387‐394. doi: 10.1016/j.endonu.2011.05.010

43. Karjalainen JJ, Kiviniemi AM, Hautala AJ, Niva J, Lepojärvi S, Mäkikallio TH, Piira OP, Huikuri HV, Tulppo MP. Effects of exercise prescription on daily physical activity and maximal exercise capacity in coronary artery disease patients with and without type 2 diabetes. *Clinical physiology and functional imaging* 2012;**32**:445‐454. doi: 10.1111/j.1475-097X.2012.01148.x

44. Kolbe-Alexander TL, Lambert EV, Charlton KE. Effectiveness of a community based low intensity exercise program for older adults. *Journal of nutrition, health & aging* 2006;**10**:21‐29. doi:

45. Stubbs EB, Fisher MA, Miller CM, Jelinek C, Butler J, McBurney C, Collins EG. Randomized controlled trial of physical exercise in diabetic veterans with length-dependent distal symmetric polyneuropathy. *Frontiers in neuroscience* 2019;**13**. doi: 10.3389/fnins.2019.00051

46. Myers J, McElrath M, Jaffe A, Smith K, Fonda H, Vu A, Hill B, Dalman R. A randomized trial of exercise training in abdominal aortic aneurysm disease. *Medicine and science in sports and exercise* 2014;**46**:2‐9. doi: 10.1249/MSS.0b013e3182a088b8

47. Paulo TRS, Rossi FE, Viezel J, Tosello GT, Seidinger SC, Simões RR, de Freitas R, Freitas IF. The impact of an exercise program on quality of life in older breast cancer survivors undergoing aromatase inhibitor therapy: a randomized controlled trial. *Health and quality of life outcomes* 2019;**17**:17. doi: 10.1186/s12955-019-1090-4

48. Pressler A, Christle JW, Lechner B, Grabs V, Haller B, Hettich I, Jochheim D, Mehilli J, Lange R, Bleiziffer S, et al. Exercise training improves exercise capacity and quality of life after transcatheter aortic valve implantation: a randomized pilot trial. *American heart journal* 2016;**182**:44‐53. doi: 10.1016/j.ahj.2016.08.007

49. Ramponi S, Tzani P, Aiello M, Marangio E, Clini E, Chetta A. Pulmonary rehabilitation improves cardiovascular response to exercise in COPD. *Respiration; international review of thoracic diseases* 2013;**86**:17‐24. doi: 10.1159/000348726

50. Rengo JL, Savage PD, Barrett T, Ades PA. Cardiac Rehabilitation Participation Rates and Outcomes for Patients With Heart Failure. *Journal of cardiopulmonary rehabilitation and prevention* 2018;**38**:38‐42. doi: 10.1097/HCR.0000000000000252

51. Stanghelle B, Bentzen H, Giangregorio L, Pripp AH, Skelton DA, Bergland A. Physical fitness in older women with osteoporosis and vertebral fracture after a resistance and balance exercise programme: 3-month post-intervention follow-up of a randomised controlled trial. *BMC musculoskeletal disorders* 2020;**21**. doi: 10.1186/s12891-020-03495-9

52. Thomas GA, Cartmel B, Harrigan M, Fiellin M, Capozza S, Zhou Y, Ercolano E, Gross CP, Hershman D, Ligibel J, et al. The effect of exercise on body composition and bone mineral density in breast cancer survivors taking aromatase inhibitors. *Obesity (Silver Spring, Md.)* 2017;**25**:346‐351. doi: 10.1002/oby.21729

53. Torres-Sánchez I, Valenza MC, Cebriá IIM, López-López L, Moreno-Ramírez MP, Ortíz-Rubio A. Effects of different physical therapy programs on perceived health status in acute exacerbation of chronic obstructive pulmonary disease patients: a randomized clinical trial. *Disability and rehabilitation* 2018;**40**:2025‐2031. doi: 10.1080/09638288.2017.1323236

54. Zaidi H, Byrkjeland R, Njerve IU, Akra S, Solheim S, Arnesen H, Seljeflot I, Opstad TB. Effects of exercise training on markers of adipose tissue remodeling in patients with coronary artery disease and type 2 diabetes mellitus: sub study of the randomized controlled EXCADI trial. *Diabetology & metabolic syndrome* 2019;**11**. doi: 10.1186/s13098-019-0508-9

55. Barcellos FC, Del Vecchio FB, Reges A, Mielke G, Santos IS, Umpierre D, Bohlke M, Hallal PC. Exercise in patients with hypertension and chronic kidney disease: a randomized controlled trial. *Journal of human hypertension* 2018;**32**:397‐407. doi: 10.1038/s41371-018-0055-0

56. Berent R, von Duvillard SP, Crouse SF, Sinzinger H, Green JS, Schmid P. Resistance training dose response in combined endurance-resistance training in patients with cardiovascular disease: a randomized trial. *Archives of physical medicine and rehabilitation* 2011;**92**:1527‐1533. doi: 10.1016/j.apmr.2011.04.021

57. Edelmann F, Gelbrich G, Düngen HD, Fröhling S, Wachter R, Stahrenberg R, Binder L, Töpper A, Lashki DJ, Schwarz S, et al. Exercise training improves exercise capacity and diastolic function in patients with heart failure with preserved ejection fraction: results of the Ex-DHF (Exercise training in Diastolic Heart Failure) pilot study. *Journal of the american college of cardiology* 2011;**58**:1780‐1791. doi: 10.1016/j.jacc.2011.06.054

58. Gary RA, Cress ME, Higgins MK, Smith AL, Dunbar SB. Combined aerobic and resistance exercise program improves task performance in patients with heart failure. *Arch Phys Med Rehabil* 2011;**92**:1371-1381. doi: 10.1016/j.apmr.2011.02.022

59. Casey D, Murphy K, Devane D, Cooney A, McCarthy B, Mee L, Newell J, O'Shea E, Scarrott C, Gillespie P, Kirwan C, Murphy AW. The effectiveness of a structured education pulmonary rehabilitation programme for improving the health status of people with moderate and severe chronic obstructive pulmonary disease in primary care: the PRINCE cluster randomised trial. *Thorax* 2013;**68**:922-928. doi: 10.1136/thoraxjnl-2012-203103

60. Nelson ME, Layne JE, Bernstein MJ, Nuernberger A, Castaneda C, Kaliton D, Hausdorff J, Judge JO, Buchner DM, Roubenoff R, Fiatarone Singh MA. The effects of multidimensional home-based exercise on functional performance in elderly people. *J Gerontol A Biol Sci Med Sci* 2004;**59**:154-160. doi: 10.1093/gerona/59.2.m154

61. Helbostad JL, Sletvold O, Moe-Nilssen R. Home training with and without additional group training in physically frail old people living at home: effect on health-related quality of life and ambulation. *Clin Rehabil* 2004;**18**:498-508. doi: 10.1191/0269215504cr761oa

62. Fisher KL, Reeder BA, Harrison EL, Bruner BG, Ashworth NL, Pahwa P, Sari N, Sheppard MS, Shields CA, Chad KE. Comparing class-based and home-based exercise for older adults with chronic health conditions: 12-month follow-up of a randomized clinical trial. *Journal of Aging and Physical Activity* 2018;**26**:471-485. doi: <http://dx.doi.org/10.1123/japa.2016-0285>

63. Bernocchi P, Vitacca M, La Rovere MT, Volterrani M, Galli T, Baratti D, Paneroni M, Campolongo G, Sposato B, Scalvini S. Home-based telerehabilitation in older patients with chronic obstructive pulmonary disease and heart failure: A randomised controlled trial. *Age and Ageing* 2018;**47**:82-88. doi: <http://dx.doi.org/10.1093/ageing/afx146>

64. Hernandez R, Cheung E, Liao M, Boughton SW, Tito LG, Sarkisian C. The Association Between Depressive Symptoms and Cognitive Functioning in Older Hispanic/Latino Adults Enrolled in an Exercise Intervention: Results From the "Caminemos!" Study. *Journal of Aging and Health* 2018;**30**:843-862. doi: <http://dx.doi.org/10.1177/0898264317696776>

65. Roitto HM, Kautiainen H, Ohman H, Savikko N, Strandberg TE, Raivio M, Laakkonen ML, Pitkala KH. Relationship of Neuropsychiatric Symptoms with Falls in Alzheimer's Disease - Does Exercise Modify the Risk? *Journal of the American Geriatrics Society* 2018;**66**:2377-2381. doi: <http://dx.doi.org/10.1111/jgs.15614>

66. Selzler AM, Rodgers WM, Berry TR, Stickland MK. The importance of exercise self-efficacy for clinical outcomes in pulmonary rehabilitation. *Rehabilitation Psychology* 2016;**61**:380-388. doi: <http://dx.doi.org/10.1037/rep0000106>

67. Shubert TE, Chokshi A, Mendes VM, Grier S, Buchanan H, Basnett J, Smith ML. Stand Tall--A Virtual Translation of the Otago Exercise Program. *Journal of Geriatric Physical Therapy* 2020;**43**:120-127. doi: 10.1519/JPT.0000000000000203

68. Toots A, Wiklund R, Littbrand H, Nordin E, Nordstrom P, Lundin-Olsson L, Gustafson Y, Rosendahl E. The Effects of Exercise on Falls in Older People With Dementia Living in Nursing Homes: A Randomized Controlled Trial. *Journal of the American Medical Directors Association* 2019;**20**:835-842.e831. doi: <http://dx.doi.org/10.1016/j.jamda.2018.10.009>

69. van Dam van Isselt EF, van Eijk M, van Geloven N, Groenewegen-Sipkema KH, van den Berg JWK, Nieuwenhuys CMA, Chavannes NH, Achterberg WP. A Prospective Cohort Study on the Effects of Geriatric Rehabilitation Following Acute Exacerbations of COPD. *Journal of the American Medical Directors Association* 2019;**20**:850-856.e852. doi: <http://dx.doi.org/10.1016/j.jamda.2019.02.025>

70. Witham MD, Daykin AR, McMurdo ME. Pilot study of an exercise intervention suitable for older heart failure patients with left ventricular systolic dysfunction. *European Journal of Cardiovascular Nursing* 2008;**7**:303-306. doi: <https://dx.doi.org/10.1016/j.ejcnurse.2008.01.109>

71. Angst F, Verra ML, Lehmann S, Benz T, Aeschlimann A. Effects of inpatient rehabilitation in hip and knee osteoarthritis: A naturalistic prospective cohort study with intraindividual control of effects. *Archives of Physical Medicine and Rehabilitation* 2013;**94**:2139-2145. doi: <http://dx.doi.org/10.1016/j.apmr.2013.03.026>

72. Gretebeck KA, Blaum CS, Moore T, Brown R, Galecki A, Strasburg D, Chen S, Alexander NB. Functional exercise improves mobility performance in older adults with type 2 diabetes: A randomized controlled trial. *Journal of Physical Activity and Health* 2019;**16**:461-469. doi: <http://dx.doi.org/10.1123/jpah.2018-0240>

73. Hessert MJ, Gugliucci MR, Pierce HR. Functional fitness: Maintaining or improving function for elders with chronic diseases. *Family Medicine* 2005;**37**:472-476. doi:

74. Lauze M, Martel DD, Aubertin-Leheudre M. Feasibility and Effects of a Physical Activity Program Using Gerontechnology in Assisted Living Communities for Older Adults. *Journal of the American Medical Directors Association* 2017;**18**:1069-1075. doi: <http://dx.doi.org/10.1016/j.jamda.2017.06.030>

75. Marengoni A, Rizzuto D, Fratiglioni L, Antikainen R, Laatikainen T, Lehtisalo J, Peltone M, Soininen H, Strandberg T, Tuomilehto J, Kivipelto M, Ngandu T. The effect of 2 year intervention of diet, physical exercise, cognitive training and monitoring of vascular risk versus control on chronic morbidity-the FINGER trial. *European Geriatric Medicine* 2017;**8 (Supplement 1)**:S12. doi:

76. Marigold DS, Eng JJ, Dawson AS, Inglis JT, Harris JE, Gylfadottir S. Exercise leads to faster postural reflexes, improved balance and mobility, and fewer falls in older persons with chronic stroke. *Journal of the American Geriatrics Society* 2005;**53**:416-423. doi: <http://dx.doi.org/10.1111/j.1532-5415.2005.53158.x>

77. Underwood M, Lamb SE, Eldridge S, Sheehan B, Slowther A, Spencer A, Thorogood M, Atherton N, Bremner SA, Devine A, et al. Exercise for depression in care home residents: a randomised controlled trial with cost-effectiveness analysis (OPERA). *Health technology assessment (Winchester, England)* 2013;**17**:1‐281. doi: 10.3310/hta17180

78. Wuthiwaropas P, Bellavia D, Omer M, Squires RW, Scott CG, Pellikka PA. Impact of cardiac rehabilitation exercise program on left ventricular diastolic function in coronary artery disease: A pilot study. *International Journal of Cardiovascular Imaging* 2013;**29**:777-785. doi: <http://dx.doi.org/10.1007/s10554-012-0152-z>

79. Lin M, Wolf SL, Hwang H, Gong S, Chen C. A randomized, controlled trial of fall prevention programs and quality of life in older fallers. *Journal of the American Geriatrics Society* 2007;**55**:499-506. doi: 10.1111/j.1532-5415.2007.01146.x

80. Robinson BS, Gordon JM, Wallentine SW, Visio M. Effectiveness of physical therapy intervention in decreasing the risk for falls in a community-dwelling aging population. *Orthopaedic Nursing* 2002;**21**:55-69. doi: 10.1097/00006416-200201000-00009

81. Suzuki T, Shimada H, Makizako H, Doi T, Yoshida D, Tsutsumimoto K, Anan Y, Uemura K, Lee S, Park H. Effects of multicomponent exercise on cognitive function in older adults with amnestic mild cognitive impairment: a randomized controlled trial. *BMC Neurology* 2012;**12**:128-128. doi: 10.1186/1471-2377-12-128

82. Brach JS, Perera S, Gilmore S, VanSwearingen JM, Brodine D, Nadkarni NK, Ricci E. Effectiveness of a Timing and Coordination Group Exercise Program to Improve Mobility in Community-Dwelling Older Adults: A Randomized Clinical Trial. *JAMA Internal Medicine* 2017;**177**:1437-1444. doi: 10.1001/jamainternmed.2017.3609

83. Stevens-Lapsley JE, Loyd BJ, Falvey JR, Figiel GJ, Kittelson AJ, Cumbler EU, Mangione KK. Progressive multi-component home-based physical therapy for deconditioned older adults following acute hospitalization: a pilot randomized controlled trial. *Clinical Rehabilitation* 2016;**30**:776-785. doi: <https://dx.doi.org/10.1177/0269215515603219>

84. Greening NJ, Williams JE, Hussain SF, Harvey-Dunstan TC, Bankart MJ, Chaplin EJ, Vincent EE, Chimera R, Morgan MD, Singh SJ, Steiner MC. An early rehabilitation intervention to enhance recovery during hospital admission for an exacerbation of chronic respiratory disease: randomised controlled trial. *BMJ* 2014;**349**:g4315. doi: <https://dx.doi.org/10.1136/bmj.g4315>

85. Pang MY, Eng JJ, Dawson AS, McKay HA, Harris JE. A community-based fitness and mobility exercise program for older adults with chronic stroke: a randomized, controlled trial. *Journal of the American Geriatrics Society* 2005;**53**:1667-1674. doi:

86. Brovold T, Skelton DA, Bergland A. Older adults recently discharged from the hospital: effect of aerobic interval exercise on health-related quality of life, physical fitness, and physical activity. *Journal of the American Geriatrics Society* 2013;**61**:1580-1585. doi: <https://dx.doi.org/10.1111/jgs.12400>

87. de Rooij M, van der Leeden M, Cheung J, van der Esch M, Hakkinen A, Haverkamp D, Roorda LD, Twisk J, Vollebregt J, Lems WF, Dekker J. Efficacy of Tailored Exercise Therapy on Physical Functioning in Patients With Knee Osteoarthritis and Comorbidity: A Randomized Controlled Trial. *Arthritis care & research* 2017;**69**:807-816. doi: <https://dx.doi.org/10.1002/acr.23013>

88. Andersson SEM, Lange E, Kucharski D, Svedlund S, Önnheim K, Bergquist M, Josefsson E, Lord JM, Mårtensson IL, Mannerkorpi K, Gjertsson I. Moderate- to high intensity aerobic and resistance exercise reduces peripheral blood regulatory cell populations in older adults with rheumatoid arthritis. *Immun Ageing* 2020;**17**:12. doi: 10.1186/s12979-020-00184-y

89. Zak M, Gryglewska B. Application of two structured rehabilitation regimens in the frail octogenarians (over 85) with functional disorders. *Rehabilitacja medyczna* 2006;**10**:20‐24. doi:

90. Gaunaurd IA, Gómez-Marín OW, Ramos CF, Sol CM, Cohen MI, Cahalin LP, Cardenas DD, Jackson RM. Physical activity and quality of life improvements of patients with idiopathic pulmonary fibrosis completing a pulmonary rehabilitation program. *Respiratory care* 2014;**59**:1872‐1879. doi: 10.4187/respcare.03180

91. Gill TM, Baker DI, Gottschalk M, Peduzzi PN, Allore H, Van Ness PH. A prehabilitation program for the prevention of functional decline: effect on higher-level physical function. *Archives of physical medicine and rehabilitation* 2004;**85**:1043‐1049. doi: 10.1016/j.apmr.2003.10.021

92. Kovács E, Prókai L, Mészáros L, Gondos T. Adapted physical activity is beneficial on balance, functional mobility, quality of life and fall risk in community-dwelling older women: a randomized single-blinded controlled trial. *European journal of physical and rehabilitation medicine* 2013;**49**:301‐310. doi:

93. Santos C, Rodrigues F, Santos J, Morais L, Bárbara C. Pulmonary Rehabilitation in COPD: effect of 2 Aerobic Exercise Intensities on Subject-Centered Outcomes--A Randomized Controlled Trial. *Respiratory care* 2015;**60**:1603‐1609. doi: 10.4187/respcare.03663

94. Morano MT, Araújo AS, Nascimento FB, da Silva GF, Mesquita R, Pinto JS, de Moraes Filho MO, Pereira ED. Preoperative pulmonary rehabilitation versus chest physical therapy in patients undergoing lung cancer resection: a pilot randomized controlled trial. *Archives of physical medicine and rehabilitation* 2013;**94**:53‐58. doi: 10.1016/j.apmr.2012.08.206

95. Kim H, Kojima N, Kim M, Yoshida H, Yoshida Y, Hirano H, Yamashiro Y, Miyanaga M, Suzuki T. Exercise and nutrition supplementation on hematological factors in community-dwelling japanese frail elderly women-RCT placebo trial. *Journal of the american geriatrics society* 2015;**63**:S32‐. doi: 10.1111/jgs.13439

96. VanSwearingen JM, Perera S, Brach JS, Wert D, Studenski SA. Impact of exercise to improve gait efficiency on activity and participation in older adults with mobility limitations: a randomized controlled trial. *Physical therapy* 2011;**91**:1740‐1751. doi: 10.2522/ptj.20100391

97. Villareal DT, Banks M, Sinacore DR, Siener C, Klein S. Effect of weight loss and exercise on frailty in obese older adults. *Archives of internal medicine* 2006;**166**:860‐866. doi: 10.1001/archinte.166.8.860

98. Yang XJ, Hill K, Moore K, Williams S, Dowson L, Borschmann K, Simpson JA, Dharmage SC. Effectiveness of a targeted exercise intervention in reversing older people's mild balance dysfunction: a randomized controlled trial. *Physical therapy* 2012;**92**:24‐37. doi: 10.2522/ptj.20100289

99. Wisniowska-Szurlej A, Cwirlej-Sozanska A, Woloszyn N, Sozanski B, Wilmowska-Pietruszynska A. Effects of physical exercises and verbal stimulation on the functional efficiency and use of free time in an older population under institutional care: a randomized controlled trial. *Journal of clinical medicine* 2020;**9**. doi: 10.3390/jcm9020477

100. Echeverria I, Amasene M, Urquiza M, Labayen I, Anaut P, Rodriguez-Larrad A, Irazusta J, Besga A. Multicomponent Physical Exercise in Older Adults after Hospitalization: a Randomized Controlled Trial Comparing Short- vs. Long-Term Group-Based Interventions. *International journal of environmental research and public health* 2020;**17**. doi: 10.3390/ijerph17020666

101. Hewitt J, Goodall S, Clemson L, Henwood T, Refshauge K. Progressive Resistance and Balance Training for Falls Prevention in Long-Term Residential Aged Care: a Cluster Randomized Trial of the Sunbeam Program. *Journal of the American Medical Directors Association* 2018;**19**:361‐369. doi: 10.1016/j.jamda.2017.12.014

102. Rubenstein LZ, Josephson KR, Trueblood PR, Loy S, Harker JO, Pietruszka FM, Robbins AS. Effects of a group exercise program on strength, mobility, and falls among fall-prone elderly men. *J Gerontol A Biol Sci Med Sci* 2000;**55**:M317-321. doi: 10.1093/gerona/55.6.m317

103. Serra-Prat M, Sist X, Domenich R, Jurado L, Saiz A, Roces A, Palomera E, Tarradelles M, Papiol M. Effectiveness of an intervention to prevent frailty in pre-frail community-dwelling older people consulting in primary care: A randomised controlled trial. *Age and Ageing* 2017;**46**:401-407. doi: <http://dx.doi.org/10.1093/ageing/afw242>

104. Matchar DB, Duncan PW, Lien CT, Ong MEH, Lee M, Gao F, Sim R, Eom K. Randomized Controlled Trial of Screening, Risk Modification, and Physical Therapy to Prevent Falls Among the Elderly Recently Discharged From the Emergency Department to the Community: The Steps to Avoid Falls in the Elderly Study. *Archives of Physical Medicine and Rehabilitation* 2017;**98**:1086-1096. doi: <http://dx.doi.org/10.1016/j.apmr.2017.01.014>

105. Reeves GR, Whellan DJ, O'Connor CM, Duncan P, Eggebeen JD, Morgan TM, Hewston LA, Pastva A, Patel MJ, Kitzman DW. A Novel Rehabilitation Intervention for Older Patients With Acute Decompensated Heart Failure: The REHAB-HF Pilot Study. *JACC: Heart Failure* 2017;**5**:359-366. doi: <http://dx.doi.org/10.1016/j.jchf.2016.12.019>

106. Baptista LC, Amorim AP, Valente-dos-Santos J, Machado-Rodrigues AM, Veríssimo MT, Martins RA. Functional status improves in hypertensive older adults: the long-term effects of antihypertensive therapy combined with multicomponent exercise intervention. *Aging Clinical & Experimental Research* 2018;**30**:1483-1495. doi: 10.1007/s40520-018-0925-x

107. Baptista LC, Machado-Rodrigues AM, Martins RA. Exercise but not metformin improves health-related quality of life and mood states in older adults with type 2 diabetes. *European Journal of Sport Science* 2017;**17**:794-804. doi: 10.1080/17461391.2017.1310933

108. Tomioka M, Braun KL, Wu YY, Holt K, Keele P, Tsuhako L, Yago J. Twelve-Month Retention in and Impact of Enhance®Fitness on Older Adults in Hawai'i. *Journal of Aging Research* 2019:1-7. doi: 10.1155/2019/9836181

109. Vahlberg B, Lindmark B, Zetterberg L, Hellström K, Cederholm T. Body composition and physical function after progressive resistance and balance training among older adults after stroke: an exploratory randomized controlled trial. *Disability & Rehabilitation* 2017;**39**:1207-1214. doi: 10.1080/09638288.2016.1191551

110. Gunther JS, Taylor MJ, Karuza J, Calkins E. Physical therapist-based group exercise/education program to improve functional health in older health maintenance organization members with arthritis. *Journal of Geriatric Physical Therapy* 2003;**26**:12-17. doi:

111. Hsieh TJ, Su SC, Chen CW, Kang YW, Hu MH, Hsu LL, Wu SY, Chen L, Chang HY, Chuang SY, et al. Individualized home-based exercise and nutrition interventions improve frailty in older adults: a randomized controlled trial. *International journal of behavioral nutrition and physical activity* 2019;**16**:119. doi: 10.1186/s12966-019-0855-9

112. Loyola WS, Camillo CA, Torres CV, Probst VS. Effects of an exercise model based on functional circuits in an older population with different levels of social participation. *Geriatrics & Gerontology International* 2018;**18**:216-223. doi: 10.1111/ggi.13167

113. Schlenk EA, Fitzgerald GK, Rogers JC, Kwoh CK, Sereika SM. Promoting Physical Activity in Older Adults With Knee Osteoarthritis and Hypertension: A Randomized Controlled Trial. *Journal of aging and physical activity* 2020:1-12. doi: <http://dx.doi.org/10.1123/japa.2019-0498>

114. Smith TP, Kennedy SL, Smith M, Orent S, Fleshner M. Physiological improvements and health benefits during an exercise-based comprehensive rehabilitation program in medically complex patients. *Exercise immunology review* 2006;**12**:86-96. doi:

115. Hinrichs T, Bucker B, Wilm S, Klaasen-Mielke R, Brach M, Platen P, Moschny A. Adverse events in mobility-limited and chronically ill elderly adults participating in an exercise intervention study supported by general practitioner practices. *Journal of the American Geriatrics Society* 2015;**63**:258-269. doi: <https://dx.doi.org/10.1111/jgs.13253>

116. Cesari M, Vellas B, Hsu FC, Newman AB, Doss H, King AC, Manini TM, Church T, Gill TM, Miller ME, et al. A physical activity intervention to treat the frailty syndrome in older persons-results from the LIFE-P study. *Journals of gerontology. Series A, Biological sciences and medical sciences* 2015;**70**:216‐222. doi: 10.1093/gerona/glu099

117. Courtney M, Edwards H, Chang A, Parker A, Finlayson K, Hamilton K. Fewer emergency readmissions and better quality of life for older adults at risk of hospital readmission: a randomized controlled trial to determine the effectiveness of a 24-week exercise and telephone follow-up program. *Journal of the American Geriatrics Society* 2009;**57**:395-402. doi: <https://dx.doi.org/10.1111/j.1532-5415.2009.02138.x>

118. Tse MM, Wan VT, Ho SS. Physical exercise: does it help in relieving pain and increasing mobility among older adults with chronic pain? *Journal of Clinical Nursing* 2011;**20**:635-644. doi: <https://dx.doi.org/10.1111/j.1365-2702.2010.03548.x>

119. Aquino G, Iuliano E, di Cagno A, Vardaro A, Fiorilli G, Moffa S, Di Costanzo A, De Simone G, Calcagno G. Effects of combined training vs aerobic training on cognitive functions in COPD: a randomized controlled trial. *International journal of chronic obstructive pulmonary disease* 2016;**11**:711‐718. doi: 10.2147/COPD.S96663

120. Boongird C, Keesukphan P, Phiphadthakusolkul S, Rattanasiri S, Thakkinstian A. Effects of a simple home-based exercise program on fall prevention in older adults: a 12-month primary care setting, randomized controlled trial. *Geriatrics & gerontology international* 2017;**17**:2157‐2163. doi: 10.1111/ggi.13052

121. Bouchonville M, Armamento-Villareal R, Shah K, Napoli N, Sinacore DR, Qualls C, Villareal DT. Weight loss, exercise or both and cardiometabolic risk factors in obese older adults: results of a randomized controlled trial. *International journal of obesity (2005)* 2014;**38**:423‐431. doi: 10.1038/ijo.2013.122

122. Campo G, Tonet E, Chiaranda G, Sella G, Maietti E, Bugani G, Vitali F, Serenelli M, Mazzoni G, Ruggiero R, et al. Exercise intervention improves quality of life in older adults after myocardial infarction: randomised clinical trial. *Heart (British Cardiac Society)* 2020. doi: 10.1136/heartjnl-2019-316349

123. Edgren J, Salpakoski A, Sihvonen SE, Portegijs E, Kallinen M, Arkela M, Jäntti P, Vanhatalo J, Pekkonen M, Rantanen T, et al. Effects of a home-based physical rehabilitation program on physical disability after hip fracture: a randomized controlled trial. *Journal of the American Medical Directors Association* 2015;**16**:350.e351‐357. doi: 10.1016/j.jamda.2014.12.015

124. Kwok BC, Pua YH. Effects of WiiActive exercises on fear of falling and functional outcomes in community-dwelling older adults: a randomised control trial. *Age and ageing* 2016;**45**:621‐627. doi: 10.1093/ageing/afw108

125. Newman AB, Dodson JA, Church T, Bonds D, Buford T, Fielding R, Kritchevsky S, Beavers DP, Pahor M, Stafford RS, et al. Cardiovascular events in a physical activity intervention as compared to a successful aging intervention: the life study randomized trial. *Circulation* 2014;**130**. doi:

126. Pérula LA, Varas-Fabra F, Rodríguez V, Ruiz-Moral R, Fernández JA, González J, Pérula CJ, Roldán AM, de Dios C. Effectiveness of a multifactorial intervention program to reduce falls incidence among community-living older adults: a randomized controlled trial. *Archives of physical medicine and rehabilitation* 2012;**93**:1677‐1684. doi: 10.1016/j.apmr.2012.03.035

127. Piva SR, Schneider MJ, Moore CG, Catelani MB, Gil AB, Klatt BA, DiGioia AM, Almeida GJ, Khoja SS, Sowa G, et al. Effectiveness of Later-Stage Exercise Programs vs Usual Medical Care on Physical Function and Activity After Total Knee Replacement: a Randomized Clinical Trial. *JAMA network open* 2019;**2**:e190018. doi: 10.1001/jamanetworkopen.2019.0018

128. Vainshelboim B, Fox BD, Kramer MR, Izhakian S, Gershman E, Oliveira J. Short-Term Improvement in Physical Activity and Body Composition After Supervised Exercise Training Program in Idiopathic Pulmonary Fibrosis. *Archives of physical medicine and rehabilitation* 2016;**97**:788‐797. doi: 10.1016/j.apmr.2016.01.018

129. Venkataraman K, Tai BC, Khoo EYH, Tavintharan S, Chandran K, Hwang SW, Phua M, Wee HL, Koh GCH, Tai ES. Short-term strength and balance training does not improve quality of life but improves functional status in individuals with diabetic peripheral neuropathy: a randomised controlled trial. *Diabetologia* 2019;**62**:2200‐2210. doi: 10.1007/s00125-019-04979-7

130. Villareal DT, Aguirre L, Gurney AB, Waters DL, Sinacore DR, Colombo E, Armamento-Villareal R, Qualls C. Aerobic or Resistance Exercise, or Both, in Dieting Obese Older Adults. *New England journal of medicine* 2017;**376**:1943‐1955. doi: 10.1056/NEJMoa1616338

131. Batra A, Palmer RC, Bastida E, McCoy HV, Khan HMR. Determining the Long-Term Effectiveness of a Group-Based Physical Activity Program. *Health promotion practice* 2019;**20**:401‐408. doi: 10.1177/1524839918769590

132. Barnett A, Smith B, Lord SR, Williams M, Baumand A. Community-based group exercise improves balance and reduces falls in at-risk older people: a randomised controlled trial. *Age Ageing* 2003;**32**:407-414. doi: 10.1093/ageing/32.4.407

133. Comans TA, Brauer SG, Haines TP. Randomized trial of domiciliary versus center-based rehabilitation: which is more effective in reducing falls and improving quality of life in older fallers? *J Gerontol A Biol Sci Med Sci* 2010;**65**:672-679. doi: 10.1093/gerona/glq054

134. Binder EF, Schechtman KB, Ehsani AA, Steger-May K, Brown M, Sinacore DR, Yarasheski KE, Holloszy JO. Effects of exercise training on frailty in community-dwelling older adults: results of a randomized, controlled trial. *J Am Geriatr Soc* 2002;**50**:1921-1928. doi: 10.1046/j.1532-5415.2002.50601.x

135. Yokochi M, Watanabe T, Ida K, Yoshida K, Sato Y. Effects of physical exercise prescribed by a medical support team on elderly lower extremity osteoarthritis combined with metabolic syndrome and/or type 2 diabetes. *Geriatrics & Gerontology International* 2012;**12**:446-453. doi: 10.1111/j.1447-0594.2011.00790.x

136. Morey MC, Cowper PA, Feussner JR, DiPasquale RC, Crowley GM, Kitzman DW, Sullivan Jr RJ. Evaluation of a supervised exercise program in a geriatric population. *Journal of the American Geriatrics Society* 1989;**37**:349-354. doi:

137. Tarazona-Santabalbina FJ, Gómez-Cabrera MC, Pérez-Ros P, Martínez-Arnau FM, Cabo H, Tsaparas K, Salvador-Pascual A, Rodriguez-Mañas L, Viña J. A Multicomponent Exercise Intervention that Reverses Frailty and Improves Cognition, Emotion, and Social Networking in the Community-Dwelling Frail Elderly: a Randomized Clinical Trial. *Journal of the American Medical Directors Association* 2016;**17**:426‐433. doi: 10.1016/j.jamda.2016.01.019

138. Kałka D, Domagała Z, Kowalewski P, Rusiecki L, Wojcieszczyk J, Kolęda P, Marciniak W, Adamus J, Janocha A, Pilecki W. The influence of endurance training intensity on dynamics of post-exertional heart rate recovery adaptation in patients with ischemic heart disease. *Advances in medical sciences* 2013;**58**:50‐57. doi: 10.2478/v10039-012-0073-z
